# Ablation of the *P21* Gene of *Trypanosoma cruzi* Provides Evidence of P21 as a Mediator in the Control of Epimastigote and Intracellular Amastigote Replication

**DOI:** 10.3389/fcimb.2022.799668

**Published:** 2022-02-18

**Authors:** Thaise Lara Teixeira, Miguel Angel Chiurillo, Noelia Lander, Cassiano Costa Rodrigues, Thiago Souza Onofre, Éden Ramalho Ferreira, Camila Miyagui Yonamine, Júlia de Gouveia Santos, Renato Arruda Mortara, Claudio Vieira da Silva, José Franco da Silveira

**Affiliations:** ^1^Departmento de Microbiologia, Imunologia e Parasitologia, Escola Paulista de Medicina, Universidade Federal de São Paulo, São Paulo, Brazil; ^2^Department of Biological Sciences, University of Cincinnati, Cincinnati, OH, United States; ^3^Laboratório de Tripanosomatídeos, Universidade Federal de Uberlândia, Uberlândia, Brazil

**Keywords:** *Trypanosoma cruzi*, Chagas disease, chronic phase, P21 protein, CRISPR/Cas9, amastigotes, proliferation, cell cycle

## Abstract

P21 is an immunomodulatory protein expressed throughout the life cycle of *Trypanosoma cruzi*, the etiologic agent of Chagas disease. *In vitro* and *in vivo* studies have shown that P21 plays an important role in the invasion of mammalian host cells and establishment of infection in a murine model. P21 functions as a signal transducer, triggering intracellular cascades in host cells and resulting in the remodeling of the actin cytoskeleton and parasite internalization. Furthermore, *in vivo* studies have shown that P21 inhibits angiogenesis, induces inflammation and fibrosis, and regulates intracellular amastigote replication. In this study, we used the CRISPR/Cas9 system for *P21* gene knockout and investigated whether the ablation of *P21* results in changes in the phenotypes associated with this protein. Ablation of *P21* gene resulted in a lower growth rate of epimastigotes and delayed cell cycle progression, accompanied by accumulation of parasites in G1 phase. However, *P21* knockout epimastigotes were viable and able to differentiate into metacyclic trypomastigotes, which are infective to mammalian cells. In comparison with wild-type parasites, *P21* knockout cells showed a reduced cell invasion rate, demonstrating the role of this protein in host cell invasion. However, there was a higher number of intracellular amastigotes per cell, suggesting that P21 is a negative regulator of amastigote proliferation in mammalian cells. Here, for the first time, we demonstrated the direct correlation between P21 and the replication of intracellular amastigotes, which underlies the chronicity of *T. cruzi* infection.

## Introduction

Chagas disease is a potentially life-threatening illness caused by the protozoan parasite *Trypanosoma cruzi*. It has been estimated that 6–7 million people are infected with *T. cruzi* worldwide, mainly in Latin America. Moreover, approximately 10,000 people die from Chagas disease each year, thus constituting an important public health problem (WHO, 2021).

The course of Chagas disease runs from an early phase with clinical symptoms or entirely without them to a latent period (often lasting for decades), which can eventually lead to severe disease. The persistence of the parasite in host tissues could be attributed to the perpetuation of the disease and its chronicity, a stage for which there is no effective treatment. Studies have reported the presence of dormant amastigotes or amastigote nests with a reduced replication rate in host tissues and how the replicative status of these parasites contributes to resistance to benznidazole therapy (Dumoulin and Burleigh, 2018; Sánchez-Valdéz et al., 2018; Ward et al., 2020). However, the mechanisms involved in these processes remain poorly understood.

*T. cruzi* circulates in the bloodstream of infected humans or reservoir mammals and invades several types of nucleated cells, within which it replicates and remains infectious. *T. cruzi* invasion and replication processes involve interactions between the parasite and host cell molecules in a coevolutionary arms race (Souza et al., 2010; Pech-Canul et al., 2017). In this framework, P21 is a protein secreted by *T. cruzi* and can be detected in the culture supernatant of parasites and in amastigotes nests present in the heart tissue of infected mice (Silva et al., 2009; Martins et al., 2020). Previous studies have shown that P21 is a key protein in the pathogenesis of Chagas disease, inducing the invasion by *T. cruzi* through activation of the chemokine receptor CXCR4, which induces the reorganization of the actin cytoskeleton promoting the parasite internalization (Silva et al., 2009; Rodrigues et al., 2012; Teixeira et al., 2017). Furthermore, recombinant P21 protein was reported to inhibit blood vessel formation by modulating the expression of angiogenesis-associated genes, inducing collagen deposition, leukocyte chemotaxis and reducing the replication of intracellular amastigotes into cardiac tissues, which could favor the permanence of the parasites inside cells and the consequent evasion of host’s immune response (Teixeira et al., 2015; Teixeira et al., 2017; Teixeira et al., 2019; Martins et al., 2020). These findings suggest a role for P21 in the chronification process of Chagas disease, in which long-term parasite persistence is determinant of the disease progression (Teixeira et al., 2015; Teixeira et al., 2019; Martins et al., 2020).

The development of molecular tools such as CRISPR/Cas9 (Doudna and Charpentier, 2014) makes it possible to precisely cleave double-stranded DNA using the Cas9 nuclease from a specific gene sequence determined by a single-guide RNA (sgRNA). The repair system allows the precise editing of the target gene, providing a multitude of uses (Knott and Doudna, 2018). For protozoan parasites, the CRISPR/Cas9 system provides the opportunity to gain new insights into parasite biology and allows the development of new approaches to study pathogenesis and parasite-host interactions at the cellular and molecular levels (Zhang et al., 2014; Sollelis et al., 2015; Lander et al., 2016; Sidik et al., 2016; Lander and Chiurillo, 2019; Young et al., 2019). Several studies have used these tools to deepen the functional understanding of *T. cruzi* and its interaction with host cells (Peng et al., 2014; Lander et al., 2015; Chiurillo et al., 2017; Lander et al., 2017; Costa et al., 2018; Akutsu et al., 2019; Chiurillo et al., 2019; Tavernelli et al., 2019; Chiurillo et al., 2020; Lander et al., 2020; Costa-Silva et al., 2021; Ibarrola-Vannucci et al., 2021, Ramakrishnan et al., 2021).

Here, we generated a *T. cruzi* knockout cell line for the *P21* gene using CRISPR/Cas9 and investigated whether *P21* deletion changes parasite phenotypes associated with the infection of mammalian host cells and the growth and replication of epimastigotes and intracellular amastigotes. Our results demonstrated the role of P21 in parasite invasion and replication within host cells and showed that CRISPR/Cas9 may be a valuable tool for *P21* gene characterization.

## Materials and Methods

### Cell Culture

*T. cruzi* epimastigotes (Y strain) were grown at 28°C in liver infusion tryptose (LIT) medium supplemented with 10% or 20% fetal bovine serum (FBS; Invitrogen) and manipulated in level 2 biosafety cabinets following institutional safety procedures. To differentiate epimastigotes into metacyclic forms, the epimastigotes were maintained in LIT medium (10% FBS) for 14 days, and metacyclic trypomastigotes were purified as previously described (Teixeira and Yoshida, 1986). Human cervical adenocarcinoma cells (HeLa cells) were cultivated in RPMI 1640 medium (Sigma-Aldrich) supplemented with 10% FBS and an antibiotic solution (10 μg/mL streptomycin, 100 U/mL penicillin, and 40 μg/mL gentamicin) in a humidified atmosphere at 37°C with 5% CO_2_.

### Determination of Selective Antibiotic Concentration

Wild-type (WT) *T. cruzi* epimastigotes (Y strain) were seeded (2 × 10^5^ cells/well) into a 96-well plate and incubated with 200 µL of LIT medium supplemented with antibiotics (G418, 150–350 µg/mL; blasticidin, 5–25 µg/mL). The parasites were kept at 28°C, and the LIT medium (containing the antibiotics) was replaced as needed. After 10 days of incubation, the number of parasites was determined by counting in a Neubauer chamber.

### Gene Knockout by CRISPR/Cas9

To knockout *P21* gene we used CRISPR/Cas9-mediated genome editing, as previously described (Lander et al., 2015; Lander et al., 2019). Briefly, the Cas9/pTREX-n vector (Addgene, catalog number: 68708) was used to clone a specific sgRNA sequence to target *P21* gene (TriTrypDB ID: TcYC6_0032830), which encodes the *T. cruzi* P21 protein.

Selection of protospacer was performed using EuPaGDT (Eukaryotic Pathogen CRISPR Guide RNA/DNA Design Tool, http://grna.ctegd.uga.edu/). This *in silico* analysis allows to choose a specific protospacer, containing a unique on-target site, and no off-targets in the genome of *T. cruzi* Y C6 TritrypDB-46 release (https://tritrypdb.org’ dataset). The protospacer sequence is shown in the **Supplementary Material**. Cas9 was expressed in fusion with human influenza virus hemagglutinin (HA) tag, SV40 nuclear localization signal (NLS), and green fluorescent protein (GFP) (Lander et al., 2015). The construct sgRNA/Cas9/pTREX-n, together with DNA donor cassettes for inducing homology-directed repair, was used to transfect *T. cruzi* epimastigotes and disrupt P21 ORF with a blasticidin resistance gene (*Bsd*; gene encoding blasticidin-S-deaminase). In addition, parasites were transfected with Cas9/pTREX-n vector containing Scrambled sgRNA sequence as previously described (Lander et al., 2015). The Scrambled protospacer sequence (GCACTACCAGAGCTAACTCA) does not recognize any target gene in the *T. cruzi* genome. All constructs were confirmed by Sanger sequencing.

The sgRNA was designed to induce a double-stranded break in the *P21* locus sequence using Cas9 nuclease. Chimeric sgRNA was obtained by PCR from plasmid pUC_sgRNA as previously described (Lander et al., 2015; Lander et al., 2019) using the oligonucleotides sgRNA Fw (**Table S1**, primer 1), which included a *Bam*HI restriction site and a 20 nt protospacer region, and sgRNA Rv (**Table S1**, primer 3), a 20 nt sequence that anneals to the sgRNA backbone. Subsequently, this sgRNA (120bp) was cloned into Cas9/pTREX-n (neomycin resistance) through the *Bam*HI site.

To generate a DNA donor cassette containing the *Bsd* gene, the pGEM-*bsd* vector was used. The template for homologous recombination was amplified by PCR using 120 bp ultramers (**Table S1**, primers 4 and 5), consisting of a 100 bp sequence corresponding to regions located upstream or downstream of the Cas9 target site of the *P21* gene, and a 20 bp sequence for annealing on the pGEM-*bsd* vector, which was used as a PCR template (Lander et al., 2015; Lander et al., 2019). PCR was carried out with the following cycling conditions (GoTaq DNA polymerase M7805; Promega): initial denaturation for 2 min at 95°C, followed by 35 cycles for 30 s at 95°C, 20 s at 63°C, and 40 s at 72°C and a final extension for 5 min at 72°C.

### Cell Transfection

*T. cruzi* epimastigotes (Y strain) in the early exponential phase were washed with phosphate-buffered saline (PBS; pH 7.4) and transfected with 25 µg of plasmid (sgRNA/Cas9/pTREX-n) diluted in P3 Primary Cell 4D-Nucleofector (V4XP-3024) solution according to the manufacturer’s instructions. The parasites were electroporated with two pulses (EH100 program) delivered by the 4D Nucleofector system (Lonza) (Cordero et al., 2019). Then, the parasites were maintained in LIT medium supplemented with 20% FBS under drug selection (250 µg/mL G418). Transfectant cell lines were sorted based on green fluorescence using the BD FACS Aria II Cell Sorter (BD Biosciences) after selection with G418, cells were electroporated with 25 µg of donor DNA, and selected with blasticidin (25 µg/mL). Next, clonal populations were selected by serial dilutions in 96-well plates. CRISPR mutant cell lines were maintained in LIT medium supplemented with 20% FBS and maintained under selection with 250 µg/mL G418 and 25 µg/mL blasticidin. The growth rate of mutant epimastigotes was determined by cell counting in a Neubauer chamber performed by two blinded observers every 48 h for 14 days.

Genomic DNA was extracted from WT, Scrambled, and knockout clones using the DNeasy Blood & Tissue Kit (69506; Qiagen) and used in PCR to verify the presence or absence of the *P21* and *Bsd* genes with specific sets of oligonucleotides (**Table S1**, primers 6–11). PCR conditions were as follows: initial denaturation for 3 min at 94°C followed by 30 cycles for 45 s at 94°C, 30 s at 60°C, and 45 s at 72°C and a final extension for 7 min at 72°C. The *P21* locus was sequenced using the Sanger method to ensure that the *Bsd* gene was inserted at the correct location (**Supplementary Material**).

### RNA Isolation and cDNA Synthesis by RT-PCR

Total RNA from WT, Scrambled, and knockout clones (1 × 10^7^ epimastigotes) was extracted using the PureLink RNA Mini Kit (12183018A; Invitrogen) and treated with DNAse I (Sigma-Aldrich) according to the manufacturer’s instructions. First-strand cDNA was synthesized from total RNA (1 µg) and oligo(dT) primers using the Superscript III First-Strand Synthesis System (18080051; Invitrogen) following the manufacturer’s instructions. Next, PCR was performed in a separate microtube using the primers listed in **Table S1** (primers 6 and 7; 12 and 13; 14 and 15), which amplify P21 and the endogenous housekeeping controls TcHGPRT (Murta et al., 2006) and TcMVK (Ferreira et al., 2016). The same PCR conditions were used to analyze the *P21* knockout clones and controls, as described in the previous section.

### Immunofluorescence Analysis of P21 Expression

WT and *P21* knockout clones were fixed with 2% paraformaldehyde and washed twice with PBS. The parasites were incubated overnight with anti-P21 polyclonal antibodies produced in mouse (1:2,000) (Silva et al., 2009) diluted in permeabilization solution (PBS pH 7.2, 0.1% gelatin, 0.1% sodium azide, and 0.2% saponin). The parasites were then incubated with anti-mouse secondary antibodies conjugated to Alexa Fluor 568 (1:200) and 4′,6-diamidino-2-phenylindole (DAPI; 1:500) for 1 h. Images were acquired in a TCS SP5 II tandem scanner (Leica) confocal microscope with a 63x NA 1.40 PlanApo oil immersion objective, and Imaris software (Bitplane) was used for imaging analysis.

### Analysis of Cell Cycle Progression by Flow Cytometry

To evaluate the cell cycle progression, WT and knockout clone parasites in log phase growth were incubated with 20 mM hydroxyurea (HU) for 18 h as previously described (Santos Júnior et al., 2021). Thus, cells were washed three times with PBS and resuspended in LIT medium (10% FBS). Samples were collected each 24 h until 120 h. At each time point parasites were washed with PBS and fixed in 500 µL methanol and PBS (1:1) at -20°C for 15 min. For DNA staining parasites were incubated with 10 µg/mL RNAse A (Invitrogen) and 10 µg/mL propidium iodide and incubated at 37°C for 30 min. Cytometry was performed on a FACSCanto II (BD Bioscience) and analyzed with the FlowJo v10 software.

### Western Blotting Analysis

Metacyclic trypomastigotes were obtained from axenic cultures of epimastigotes in the stationary phase maintained in LIT medium (pH 7.2) for 14 days at 28°C. After differentiation, the number of parasites was counted in a Neubauer chamber. Then, the samples were washed twice with PBS, and the pellet was resuspended in 50 µL of lysis buffer (10 mM Tris-HCl pH 7.5, 50 mM NaCl, 5 mM EDTA, 4 mM Na_3_VO_4_, 2 mM NaF, 20% glycerol, 1% NP-40, and complete-C protease inhibitors). The extracts were incubated on ice for 30 min and centrifuged at 13,000 × *g* for 10 min at 4°C. The supernatant was recovered and frozen at -20°C.

The protein concentration of the parasite extracts was estimated by Bradford assay, and 40 µg of total protein was loaded onto a 10% SDS-PAGE gel and transferred to a nitrocellulose membrane. The gp82 and α-tubulin proteins were labeled using the monoclonal antibodies 3F6 (1:1,500) (Teixeira and Yoshida, 1986) and anti-α-tubulin (1:2,500; Sigma-Aldrich), respectively, both of which were produced in mice. The membranes were blocked with 5% skimmed milk powder in 0.1% PBS-T solution (PBS containing 0.1% Tween 20) for 1 h under constant agitation at room temperature. After blocking, the membranes were washed three times with PBS-T 0.1% for 10 min and incubated with antibodies against gp82 and α-tubulin, followed by anti-mouse IgG-peroxidase antibodies (1:6,000) in the same blocking solution; the membranes were washed three times before each incubation. Protein bands were detected using Immobilon Western Chemiluminescent HRP Substrate (Millipore) and Amersham Hyperfilm™ ECL (GE Healthcare). The intensity of the bands associated with the gp82 and α-tubulin proteins was quantified using GelAnalyzer 19.1 software; the density of each band was converted to peaks, and the area under the peaks was used to determine the pixel intensity. The assays were performed in two independent experiments.

### Invasion and Replication Assays

HeLa cells were seeded onto 24-well plates containing 13 mm coverslips to a final density of 1 × 10^5^ cells/well for invasion and 5 × 10^4^ cells/well for replication assays and maintained overnight under optimal conditions for adhesion. Then, the cells were incubated for 2h with purified metacyclic forms of the WT and *P21* knockout clones at a multiplicity of infection (MOI) of 20 (20 parasites:1 host cell) for invasion and 10 (10 parasites:1 host cell) for replication.

The cells were washed three times with PBS to remove non-internalized parasites, fixed with Bouin’s solution (HT10132; Sigma-Aldrich) for 5 min, washed with PBS, and stained with Giemsa (1:3) (GS500; Sigma-Aldrich) for 1 h. Then, the stained cells were dehydrated sequentially in acetone, acetone:xylol, and xylol and mounted on slides with Entellan^®^ (Merck Millipore), and the number of infected cells was quantified for 100 cells.

For the replication assays, after 2 h, HeLa cells were washed and maintained in optimal conditions for 24h, 48h or 72 h. Then, the cells were fixed with 2% paraformaldehyde and incubated with anti-chagasic human serum (1:100) diluted in permeabilization solution overnight. The cells were washed with PBS and incubated with secondary antibodies conjugated to Alexa Fluor 568 (1:200) and DAPI (1:500) for 1 h. The number of parasites per cell was quantified for 100 infected cells. Representative images of intracellular amastigotes were captured using a confocal microscope (Leica SP5), and Imaris software (Bitplane) was used to perform the analysis.

### Statistical Analysis

Statistical analyses were performed using GraphPad Prism software version 7.0. All values are expressed as the mean ± standard deviation of two independent assays performed at least in duplicate. Differences were considered significant at *p* < 0.05. Significant differences were determined by one-way ANOVA and Tukey’s test for multiple comparisons of parametric data, Kruskal-Wallis and Dunn’s test for multiple comparisons of nonparametric data, or two-way ANOVA and Tukey’s or Sidak’s test for multiple comparisons.

## Results

### *T. cruzi P21* Gene Knockout Strategy

In this work, gene P21 from strain Y clone C6 (Y C6) (Gene ID: TcYC6_0032830) was selected for functional analysis. The genome of Y C6 was organized into 40 chromosome-scale scaffolds, many of which represent near-complete chromosomes, such as the chromosome 17 where gene P21 has been assigned (Wang et al., 2021). The gene *P21* encodes a 154-amino acid protein (TcYC6_0032830-RA) and it was mapped (position:58.538-59.002 nt) near one end of chromosome 17. *P21* is located within a polycistronic unit of about 230,000 nt belonging to a syntenic region conserved in different strains of *T. cruzi*. Nucleotide blast analysis demonstrate that P21 protein is coded by a pair of alleles and no orthologs were found in other trypanosomatids (https://tritrypdb.org/tritrypdb/app/record/gene/TcYC6_0032830).

Firstly, we determined the antibiotic concentration of G418 and blasticidin for selecting the epimastigotes of the Y strain under culture conditions. They were incubated with different concentrations of antibiotics for 10 days, and the number of parasites was estimated by counting in a Neubauer chamber. We found that 250 µg/mL G418 and 25 µg/mL blasticidin could be used to select the parasites after electroporation (**Supplementary Figures 1A, B**).

To obtain *P21* knockout clones, we used a single vector for the co-expression of Cas9 and sgRNA. As demonstrated in a previous study, this sgRNA/Cas9/pTREX-n construct can induce the transcription of Cas9 and sgRNA and the translation of Cas9 alone with nuclear import (Cas9-HA-2xNLS-GFP) (Lander et al., 2015). The *P21* sgRNA was cloned into the Cas9/pTREX-n vector, which was confirmed by Sanger sequencing (**Supplementary Figure 1C**). We also cloned a non-targeting (Scrambled) sgRNA into the Cas9/pTREX-n vector as the control. Subsequently, donor DNA (599 bp) was synthesized by PCR using ultramer primers (which contain the homologous recombination sequences plus 20 nt that recognizes the *Bsd* gene) and the pGEM-*Bsd* vector as a template. The plasmids were used to electroporate epimastigotes, generating lines that express the Cas9-GFP fusion protein in the parasite nucleus (**Supplementary Figure 1D**). After the selection with G418 (250 µg/mL), the parasites were electroporated with donor DNA (**Supplementary Figure 1E**), which contained the “homology arms” and the *Bsd* gene, and selected with 25 µg/mL blasticidin and 250 µg/mL G418.

**Figure 1:**
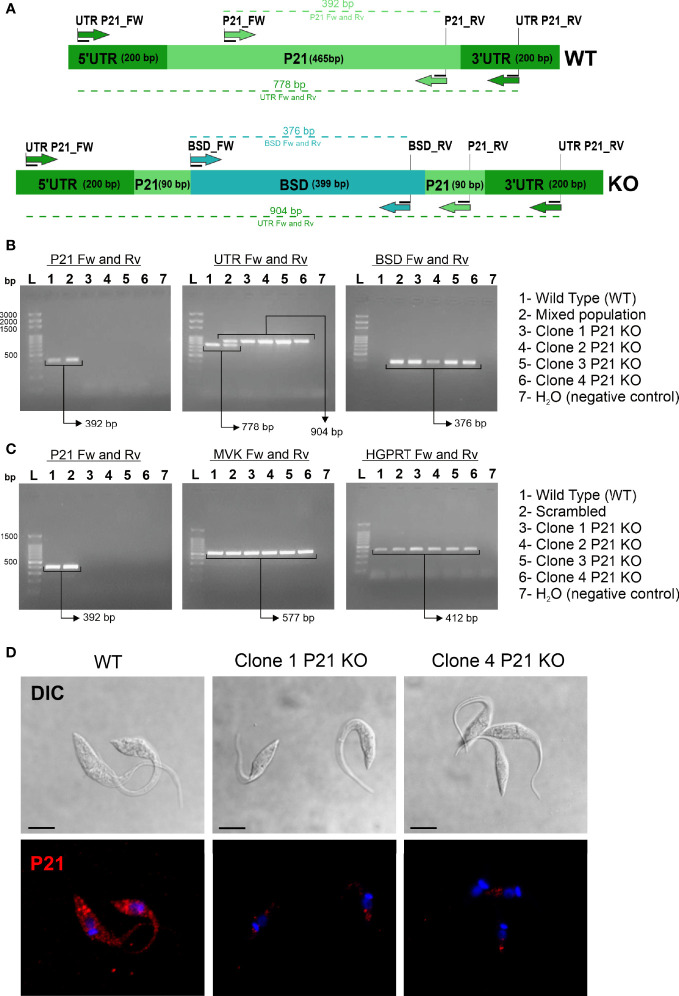
Confirmation of *P21* knockout clones. **(A)** Schematic representation of the *P21* gene locus in WT and knockout clones. The indicated primers are shown in **Table S1**. **(B)** PCR of gDNA isolated from knockout clones and controls (WT and Scrambled) using specific primers for *P21*, the UTR region of the *P21* gene, and the blasticidin resistance gene (*Bsd*). **(C)** PCR of cDNA from knockout clones and controls (WT and Scrambled) by analyzing the expression of *P21* and endogenous TcMVK and TcHGPRT. **(D)** Epimastigotes were incubated with polyclonal anti-P21 antibodies (mouse) and anti-mouse antibodies conjugated to Alexa Fluor 568 (red). The nucleus and kinetoplast were labeled with DAPI (blue). DIC, differential interference contrast; P21 KO, *P21* knockout. Single plane images were acquired by confocal microscopy. The scale bars represent 5 µm.

After antibiotic selection, the genomic DNA of transfected parasites was extracted and analyzed by PCR with primers that anneal in the *P21*, *Bsd*, and *P21* UTR regions (**Tables S1**, **S2**). The gel electrophoresis profile of amplicons found on the transfected population (sgRNA_p21) indicated the presence of both the *P21* and *Bsd* genes, suggesting a mixed population (**Supplementary Figure 2**). The mixed population was cloned by limiting dilution at 0.2 parasites/well. We isolated four *P21* knockout clones, which were analyzed by PCR. **Figure 1A** shows a schematic representation of the *P21* WT and *P21* knockout gene *locus* and the alignment sites of the primers used to screen for knockout clones. We found that DNA from the WT and mixed populations showed amplification of the *P21* gene (392 bp); while the four knockout clones did not show amplification (**Figure 1B**). Furthermore, when we analyzed the amplicons obtained using the UTR primers, we identified a 778 bp band from the WT control and a 904 bp band from the knockout clones. The mixed population showed both bands, suggesting the deletion of a single *P21* allele in this population.

In addition, we analyzed the *Bsd* insertion site in the 4 knockout clones by PCR using the combination of UTR Fw/BSD Rv, and BSD Fw/UTR Rv primers. The results demonstrate that the *Bsd* gene was inserted between the UTR regions of the P21 gene (**Supplementary Figure 3**). Furthermore, to confirm whether the *Bsd* sequence was correctly inserted in the *P21 locus*, amplicons obtained by PCR using UTR primers were purified and sequenced, and the results indicated that homologous recombination with the *Bsd* gene was successful (**Supplementary Material_sequencing**).

### Analysis of *P21* Gene Expression in Knockout Clones

To confirm the absence of *P21* transcripts in the *P21* knockout clones, we analyzed cDNA clones synthesized by RT-PCR from total RNA using primers for the *P21* coding sequence. Two housekeeping genes were included as internal controls for the assay: *T. cruzi* mevalonate kinase (TcMVK) (Ferreira et al., 2016) and hypoxanthine-guanine phosphoribosyltransferase (TcHGPRT) (Murta et al., 2006). The results demonstrated that there were no *P21* transcripts in the knockout clones. On the other hand, all clones expressed TcMVK and HGPRT transcripts (**Figure 1C**). We also analyzed the expression of the P21 protein in parasites by immunofluorescence assay. The parasites were labeled with mouse anti-P21 primary antibodies and anti-mouse secondary antibodies conjugated to Alexa Fluor 568 and analyzed by confocal microscopy. In WT epimastigotes, the P21 protein was clustered at multiple points; however, knockout parasites showed a weak labeling, a nonspecific trace of detection from the polyclonal antibody (**Figure 1D**).

### Effects of *P21* Ablation on Epimastigote Growth and Cell Cycle Progression

**Figure 2:**
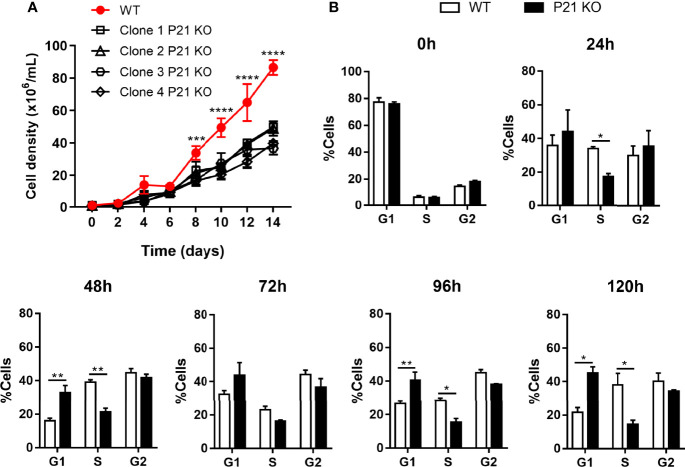
Effects of *P21* ablation on epimastigote growth and cell cycle progression. **(A)** Epimastigote growth curve for 14 days. The graph shows the representative mean ± SD of one of the two independent experiments performed in quadruplicate. The comparison was performed by two-way ANOVA and Tukey’s test for multiple comparisons **(B)** Flow Cytometry analysis of the cell cycle progression, after treatment with hydroxyurea for 18 h. The graphs represent a percentage of parasites in each cell cycle phase per time point (24-120 h). The first graph (0h) represents the synchronization of the parasites in the G1 phase. Data represent the mean ± SD of one of two independent experiments performed in triplicate. The comparison was performed by two-way ANOVA and Sidak’s test for multiple comparisons. Asterisks indicate significant differences. P21 KO, *P21* knockout. *p* value: * < 0.05, ** < 0.01, *** < 0.001, **** < 0.0001.

To investigate whether P21 deficiency hinders the growth of *T. cruzi*, we evaluated the growth curve of *P21* knockout clones compared with that of WT parasites for 14 days by counting the number of parasites every 2 days. We found that *P21* knockout cells exhibit a slower growth rate than WT parasites (**Figure 2A**). We also compared the growth curve between WT, Scrambled and clone 1 P21 KO. The data showed that the profile of the curve is similar between WT and Scrambled even though at the times of 10 and 14 days there was a statistical difference. But, similarly to what was found previously, between the 8th and 14th day, clone 1 P21 KO showed reduced growth rate compared to WT and Scrambled controls (**Supplementary Figure 4**).

Furthermore, we examined the cell cycle progression (24-120 h) of WT and *P21* knockout parasites by flow cytometry after synchronization cells with hydroxyurea treatment. The first graph (0H) demonstrated that most of the parasites from both groups were synchronized in the G1 phase of the cell cycle. At other times the data demonstrated that *P21* ablation altered the cell cycle progression compared with WT parasites. *P21* knockout parasites accumulated in G1 and decreased in S phase throughout the cell cycle while WT cells presented normal progression (**Figure 2B**). These data indicate that *P21* ablation impairs epimastigote replication.

**Figure 3:**
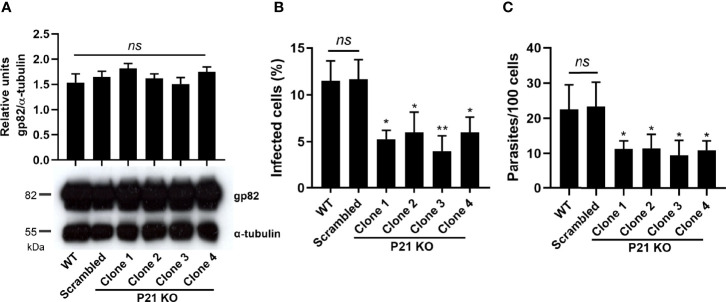
Effects of *P21* ablation on metacyclic forms and its invasion in HeLa cells. **(A)** Western blot and densitometry analysis of WT, Scrambled, and knockout clones. Antibodies for detecting gp82 and α-tubulin (50 kDa, loading control) were used. Densitometry was performed by normalizing the relative expression of gp82 to the control expression, and the graph represents the mean ± SD of the representative values of one assay performed in duplicate. **(B)** Percentage of infected HeLa cells after 2 h of invasion with purified metacyclic trypomastigotes. **(C)** Number of internalized parasites. The graphs show the representative mean ± SD of one of the two independent experiments performed in triplicate. The comparison was performed by one-way ANOVA and Tukey’s test for multiple comparisons. Asterisks indicate significant differences. P21 KO, *P21* Knockout; ns, no significant differences. *p* value: *< 0.05, **< 0.01.

We also investigated the effect of *P21* ablation in *T. cruzi* metacyclogenesis. We analyzed the expression of the glycoprotein gp82, a molecular marker of the differentiation of epimastigotes into metacyclic trypomastigotes, after 14 days of growth. The results showed that there was no difference in the expression of gp82 between the knockout clones and controls (**Figure 3A**).

### Effects of *P21* Ablation on Metacyclic Trypomastigote Invasion and Intracellular Amastigote Replication

Previous studies have shown that P21 plays an important role in the *T. cruzi* invasion process. Therefore, we investigated whether the absence of *P21* transcripts affects the invasion of host cells. We infected HeLa cells with purified metacyclic trypomastigotes for 2 h and analyzed the rate of invasion and the number of internalized parasites by counting 100 cells. *P21* knockout cells showed a significant reduction in the invasion rate compared with that of WT or Scrambled controls (**Figures 3B, C**).

**Figure 4:**
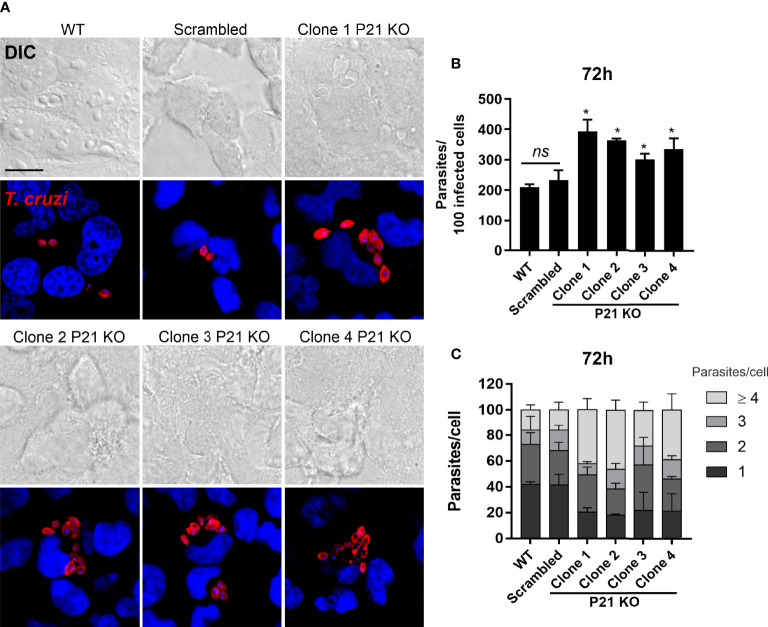
Effects of *P21* ablation on the replication of intracellular amastigotes in HeLa cells. **(A)** Representative confocal microscopy images showing the intracellular replication of amastigotes in HeLa cells after 72 h of invasion. Amastigotes were incubated with chagasic serum and anti-human antibodies conjugated to Alexa Fluor 568 (red). The nucleus and kinetoplast were labeled with DAPI (blue). DIC, differential interference contrast. Single plane images were acquired by confocal microscopy. The scale bar represents 20 µm. **(B)** Total number of parasites in 100 infected cells. The graph shows the representative mean ± SD of one of the two independent tests performed in triplicate. **(C)** Number of parasites per cell for 100 infected cells. A total of 100 single-infected cells were analyzed per assay. The bars represent the mean ± SD of two independent experiments performed in triplicate. The comparison was performed by one-way ANOVA and Tukey’s test for multiple comparisons. Asterisks indicate significant differences. P21 KO, *P21* Knockout; ns, no significant differences. *p* value: *< 0.05.

We also investigated the replication pattern of the parasites at 24, 48 and 72 hours after invasion by counting the parasites present in 100 infected cells. The results showed that at the times of 24 and 48 hours there was no difference in replication between the analyzed groups (**Supplementary Figure 5**). However, at the time of 72 hours after invasion the overall replication rate of knockout clones was higher than that of WT and Scrambled parasites (**Figures 4A, B**). Furthermore, we determined the number of parasites per cell for 100 infected cells, and there was a larger number of cells with ≥ 4 parasites among cells infected with knockout clones compared with those infected with controls, as shown in **Figure 4C**. The findings indicated that *P21* ablation impaired metacyclic trypomastigotes invasion and increased the replication rate of intracellular amastigotes.

## Discussion

Bioinformatics analysis of the genome of P21 in different strains of *T. cruzi* has revealed that the P21 protein is encoded by a single-copy gene, and no orthologs are found in other trypanosomatids. The Y strain (lineage TcII) is homozygous for the *P21* locus, which has been assigned to one end of the *in silico* assembled chromosome 17 of the *T. cruzi* Y C6 strain (TriTrypDB ID: TcYC6_0032830). In this study, we used the CRISPR/Cas9 system, a robust genetic tool, to investigate the biological roles of the P21 protein. We obtained four double-knockout clones, which the *P21* gene *locus* was sequenced, confirming the insertion of the *Bsd* gene. No other rearrangements were identified at this particular *locus*.

We evaluated the growth and differentiation phenotypes of epimastigotes after *P21* ablation. First, we determined the growth curve of knockout clones and WT control for 14 days. From day 8 onwards, knockout clones grew slower than WT parasites, and on day 14, their growth rate was around twice slower than that of WT epimastigotes. Interestingly, this result showed that *P21* gene knockout impaired the growth of epimastigotes. We also analyzed the cell cycle progression after synchronization of cells with hydroxyurea treatment. The results showed that *P21* ablation induces an increase in the number of parasites in the G1 and S phases of the cycle at most analyzed times, indicating a delayed cell cycle progression, which corroborates the reduced growth of epimastigotes observed. A previous study reported that the incubation of epimastigotes for 72 h with recombinant P21 protein (rP21) caused an increase in the number of G1 and S phase parasites with no interference in the G2 phase of the cell cycle (Teixeira et al., 2019). This discrepancy may be attributed to methodological differences, selective pressure, or the study time interval and needs to be further investigated.

*T. cruzi* epimastigotes survive solely in the gastrointestinal tract of the insect vector where a vigorous immune response may not be mounted in order to avoid eliminating obligate symbiotic microbes on which they rely for survival (Salcedo-Porras and Lowenberger, 2019). In this context, the stressful condition that triggers epimastigote forms differentiation into metacyclic trypomastigotes is parasite starvation. Martins et al., 2020 demonstrated that epimastigotes undergoing nutritional starvation increased P21 expression. Here we observed that *P21* knockout parasites showed a lower growth rate compared to controls. This data suggests that P21 somehow plays a role in epimastigote multiplication. If P21 is more expressed during parasite starvation, it is reasonable to speculate that the protein has also an impact on parasite metacyclogenesis. The expression of gp82 was not a good parameter to determine the metacyclogenesis process, since we observed similar levels of gp82 among the groups of parasites. This may be explained by the fact that gp82 is also expressed by intermediate forms of the parasite, as shown by Bayer-Santos et al., 2013. From this, the direct impact of P21 over epimastigote differentiation or an indirect effect due to the lower multiplication rate (lower starvation), will be further studied in future investigations.

As P21 plays an important role in the invasion of mammalian cells by trypomastigotes (Silva et al., 2009; Rodrigues et al., 2012), we also investigated whether the absence of *P21* transcripts affects the invasion process. We determined the rate of invasion by metacyclic trypomastigotes by counting the number of HeLa cells infected with parasites. In comparison with WT and Scrambled controls, knockout clones showed a significant reduction in the invasion rate and the number of internalized parasites.

*T. cruzi* invasion involves surface and secreted parasite molecules that act through complex signaling pathways and trigger cellular responses, leading to the invasion of host cells (reviewed in Caradonna and Burleigh, 2011). Previous studies found that P21 could induce the entry of parasites into HeLa cells and murine macrophages by acting as a signal transducer, triggering intracellular cascades in host cells and resulting in actin cytoskeleton remodeling and parasite internalization through its binding to the CXCR4 receptor (Silva et al., 2009; Rodrigues et al., 2012). Consistent with these findings, we demonstrated that *P21* deletion affected the entry of metacyclic forms of the parasites into HeLa cells, decreasing the percentage of infected cells by half and reducing the number of internalized parasites.

It has been reported that rP21 inhibits the intracellular replication of amastigotes by regulating the multiplication of the parasites in host cells (Teixeira et al., 2019; Martins et al., 2020). To investigate whether *P21* deletion affects the replication of amastigotes, HeLa cells were infected with *P21* knockout metacyclic trypomastigotes, and after 24, 48 or 72 h of growth, the infected cells were immunostained with anti-*T. cruz*i antibodies and counterstained with DAPI. We estimated the number of parasites present in each cell from a sample of 100 infected host cells. The proportion of host cells with ≥ 4 parasites was higher among cells infected with knockout clones compared with those infected with WT and Scrambled controls. In agreement with previous findings, *P21* gene knockout produced a result opposite to the inhibitory effect of rP21 in the replication of amastigotes. Taken together, the lack of P21 expression decreased metacyclic trypomastigote cell invasion and increased intracellular amastigote multiplication in a way consistent with our previous data using the recombinant form of P21. Moreover, we believe that the discrepancy concerning the impact of P21 expression in epimastigote and intracellular amastigote multiplication may be due to different signaling events triggered by the different parasite forms. Epimastigote and amastigote are the replicative forms of the parasite. However, many morphological and biochemical differences exist between these evolutionary forms. Epimastigotes infect the gastrointestinal tract of the insect vector and suffer from starvation in order to differentiate into metacyclic trypomastigotes. Amastigotes are found free in the cytosol of mammalian host cell, faces different sources of stressors, differentiates into bloodstream trypomastigote and is responsible for the perpetuation of the infection. These different conditions of life permitted us to envisage that different molecular partners are triggered during P21 signaling pathway in epimastigote and amastigote replication process.

Several studies have examined whether the presence of *T. cruzi* in dormant or low-replication states is related to persistence in host tissues over several years and whether its ability to remain in these states is related to resistance to the drugs used in the treatment of Chagas disease. Dumoulin and Burleigh (2018) demonstrated that intracellular amastigotes use a strategy referred to as growth plasticity to adapt to and recover from environmental stressors (e.g., nutrient deprivation, metabolism blockers, or exposure to benznidazole). Another study demonstrated that the key to the resistance of *T. cruzi* lies in non-replicating or dormant amastigotes that appear heterogeneously in host cells and resist drug treatment, which may later multiply and generate trypomastigotes capable of invading new cells (Sánchez-Valdéz et al., 2018). On the other hand, Ward et al. (2020) reported that there are more amastigotes in the S phase of the cell cycle in the acute phase than in the chronic phase. Therefore, the parasites can respond to environmental pressure and reduce their replication rate. Furthermore, the authors raised the possibility that the parasites may enter quiescence in the presence of benznidazole due to DNA damage caused by the drug.

Martins et al., 2020 showed that the treatment of C2C12 myoblasts with rP21 reduced the replication rate of intracellular amastigotes and induced an increase in actin polymerization in host cells. Furthermore, mice infected with *T. cruzi* and treated with rP21 exhibited a reduced parasite load in cardiac tissues (Teixeira et al., 2019; Martins et al., 2020). The authors also reported the presence of native P21 secreted by amastigote nests located in the cardiac tissues of infected mice. In addition, intracellular amastigotes were found to express higher levels of P21 when host cells were treated with IFN-γ (Martins et al., 2020). One of the possible explanations for these findings is that P21 may be a pleiotropic protein secreted during parasite cell invasion and acting as a modulator of parasite replication. These attributes may confer the parasite the ability to establish chronic infection and resistance to stress conditions from the mammalian host. The mechanisms beneath these modulation properties will be better studied.

In this work we have provided evidence demonstrating the specific disruption of the TcP21 locus in KO cell lines following a CRISPR/Cas9 strategy that has been successfully used in several studies (reviewed in Lander et al., 2019; Chiurillo and Lander, 2021). To further investigate the physiological function of TcP21 and its role in host cell infection, complementation studies should be performed by restoring the WT P21 gene in KO parasites to confirm and validate our results.

In conclusion, this study demonstrated that P21 ablation could affect epimastigote cell cycle progression, reduce parasite cell invasion, and increase intracellular amastigote replication. Taken together, the results suggest that P21 may play a role in the infectivity of metacyclic trypomastigotes and in the replication rate of amastigotes, which could be crucial for the establishment and perpetuation of infection.

## Data Availability Statement

The original contributions presented in the study are included in the article/**Supplementary Material**. Further inquiries can be directed to the corresponding authors.

## Author Contributions

TT, MC, NL, CS, and JFS conceived and designed experiments. TT, MC, NL, CR, TO, ÉF, CY, and JGS performed experiments. TT and JFS wrote the manuscript. TT prepared the figures. TT, JFS, CS, and MC discussed the results. All authors reviewed the manuscript. All authors contributed to the article and approved the submitted version.

## Funding

This study was supported by grants and fellowships from FAPESP, FAPEMIG, CAPES, and CNPq. FAPESP process numbers: 2016/15000-4 (Thematic project) and 2019/05049-4 (Postdoctoral fellowship); FAPEMIG process number: APQ-00971-17.

## Conflict of Interest

The authors declare that the research was conducted in the absence of any commercial or financial relationships that could be construed as a potential conflict of interest.

## Publisher’s Note

All claims expressed in this article are solely those of the authors and do not necessarily represent those of their affiliated organizations, or those of the publisher, the editors and the reviewers. Any product that may be evaluated in this article, or claim that may be made by its manufacturer, is not guaranteed or endorsed by the publisher.

## Supplementary Material

Supplementary Figure 1 Strategy for obtaining *P21* knockout clones. **(A, B)** Number of wild type Y strain parasites after 10 days of incubation with the antibiotics G418 and blasticidin. The graph represents the mean ± SD of two independent experiments performed in duplicate. Asterisks indicate statistical significance. The comparison was performed by Kruskal-Wallis test and Dunn’s test for multiple comparisons. *p* value: *< 0.05, **< 0.01. **(C)** Representative image of the sequencing of the *P21* sgRNA cloned in pTREX/Cas9. P21_PS_RC represents the sequence of protospacer (query); Seq_2-9-1 and Seq_2-9-2 represent the sequence cloned into a vector, obtained from sequencing, performed in duplicate. **(D)** Representative images of the fluorescence microscopy of parasites electroporated with sgRNA expressing Cas9-EGFP. **(E)** Donor DNA (599 bp) obtained by PCR using ultramer primers (**Table S1**) and the pGEM-*Bsd* vector as a template.

Supplementary Figure 2 PCR profile of amplicons obtained from a mixed population. **(A)** Schematic representation of the *P21* gene locus in WT and knockout clones. All primer combinations used as well as amplicon sizes are shown in the figure. **(B)** Agarose gels show amplicons obtained by PCR of the WT and sgRNA_*P21* populations using different sets of primers. The results indicated a mixed population, with hemi- or double-knockout parasites prior to cloning by limiting dilution. The schematic representation in **Figure 2** shows the sites of primer annealing, and **Table S2** lists all possible amplicons that can be obtained with the different primer combinations. WT, wild type; sgRNA_*P21*, parasites transfected with sgRNA and DNA donor before cloning (mixed population).

Supplementary Figure 3 Confirmation of insertion of the *Bsd* gene within the P21 UTR in knockout clones. Agarose gel shows amplicons obtained by PCR of the WT, Scrambled and 4 knockout clones using UTR Fw/BSD Rv and BSD Fw/UTR Rv primers combination.

Supplementary Figure 4 Growth curve of epimastigotes comparing WT, Scrambled and Clone 1 P21 KO groups. Epimastigote growth curve for 14 days comparing WT, Scrambled and Clone 1 P21 KO parasites. The graph shows the representative mean ± SD of one experiment performed in sextuplicate. The comparison was performed by two-way ANOVA and Sidak’s test for multiple comparisons. *indicated a statistical difference between WT and Scrambled (p < 0,05). ^#^indicated a statistical difference between WT, Scrambled and Clone 1 P21 KO.

Supplementary Figure 5 Replication of intracellular amastigotes in HeLa cells after 24 and 48 hours of invasion. Total number of parasites in 100 infected cells. The graph shows the representative mean ± SD of one experiment performed in quintuplicate. The comparison was performed by Kruskal-Wallis test and Dunn’s teste for multiple comparison. ns, no significant differences.
