# Supplementary figures and images for: Ablation of the P21 Gene of Trypanosoma cruzi Provides Evidence of P21 as a Mediator in the Control of Epimastigote and Intracellular Amastigote Replication
Source: Front Cell Infect Microbiol. 2022 Feb 18;12:799668. doi: 10.3389/fcimb.2022.799668 (PMC8895596; doi:10.3389/fcimb.2022.799668)

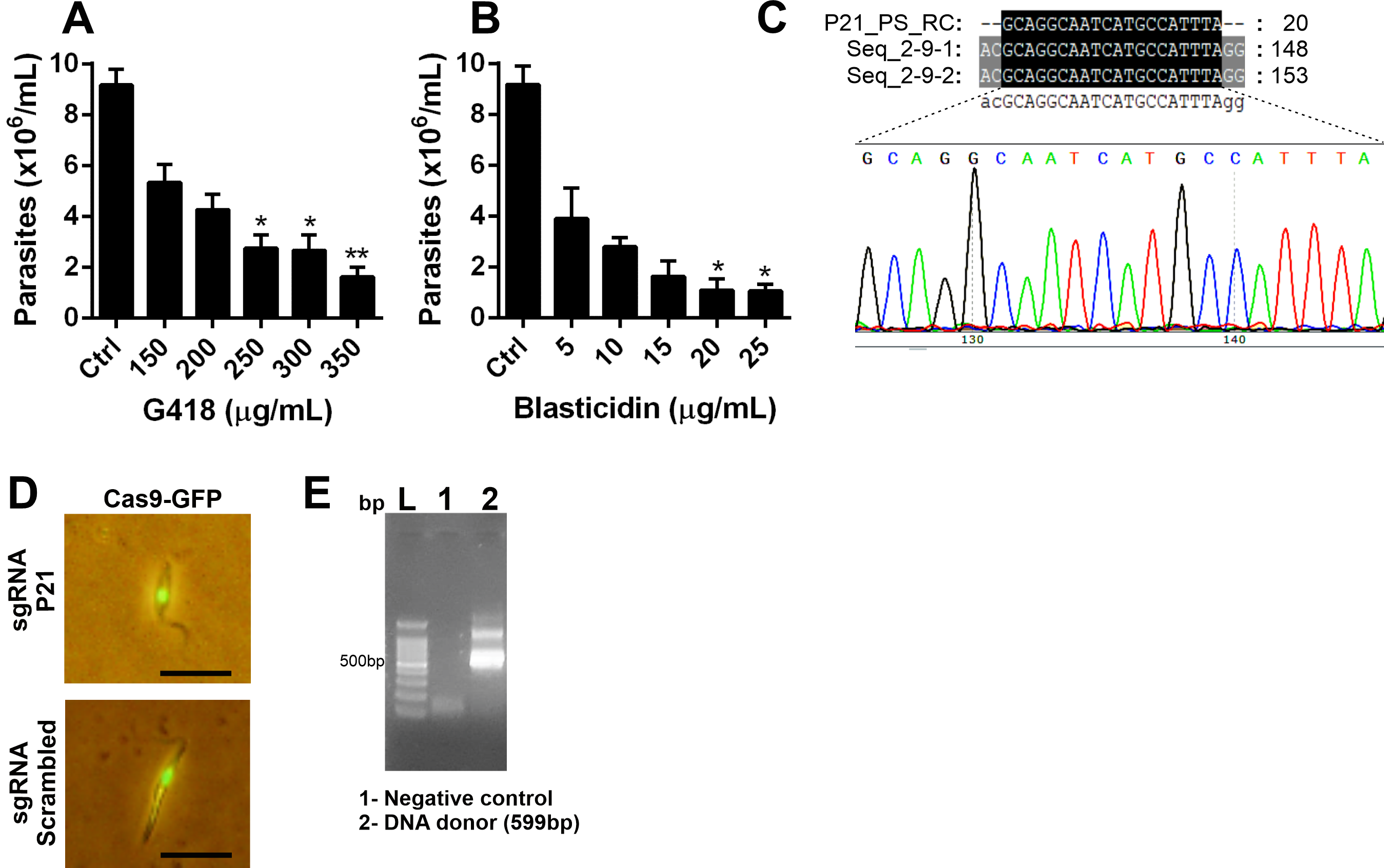

Supplement: Supplementary Figure 1 — Strategy for obtaining P21 knockout clones. (A, B) Number of wild type Y strain parasites after 10 days of incubation with the antibiotics G418 and blasticidin. The graph represents the mean ± SD of two independent experiments performed in duplicate. Asterisks indicate statistical significance. The comparison was performed by Kruskal-Wallis test and Dunn’s test for multiple comparisons. p value: *< 0.05, **< 0.01. (C) Representative image of the sequencing of the P21 sgRNA cloned in pTREX/Cas9. P21_PS_RC represents the sequence of protospacer (query); Seq_2-9-1 and Seq_2-9-2 represent the sequence cloned into a vector, obtained from sequencing, performed in duplicate. (D) Representative images of the fluorescence microscopy of parasites electroporated with sgRNA expressing Cas9-EGFP. (E) Donor DNA (599 bp) obtained by PCR using ultramer primers (Table S1) and the pGEM-Bsd vector as a template. [file Image_1.tif]

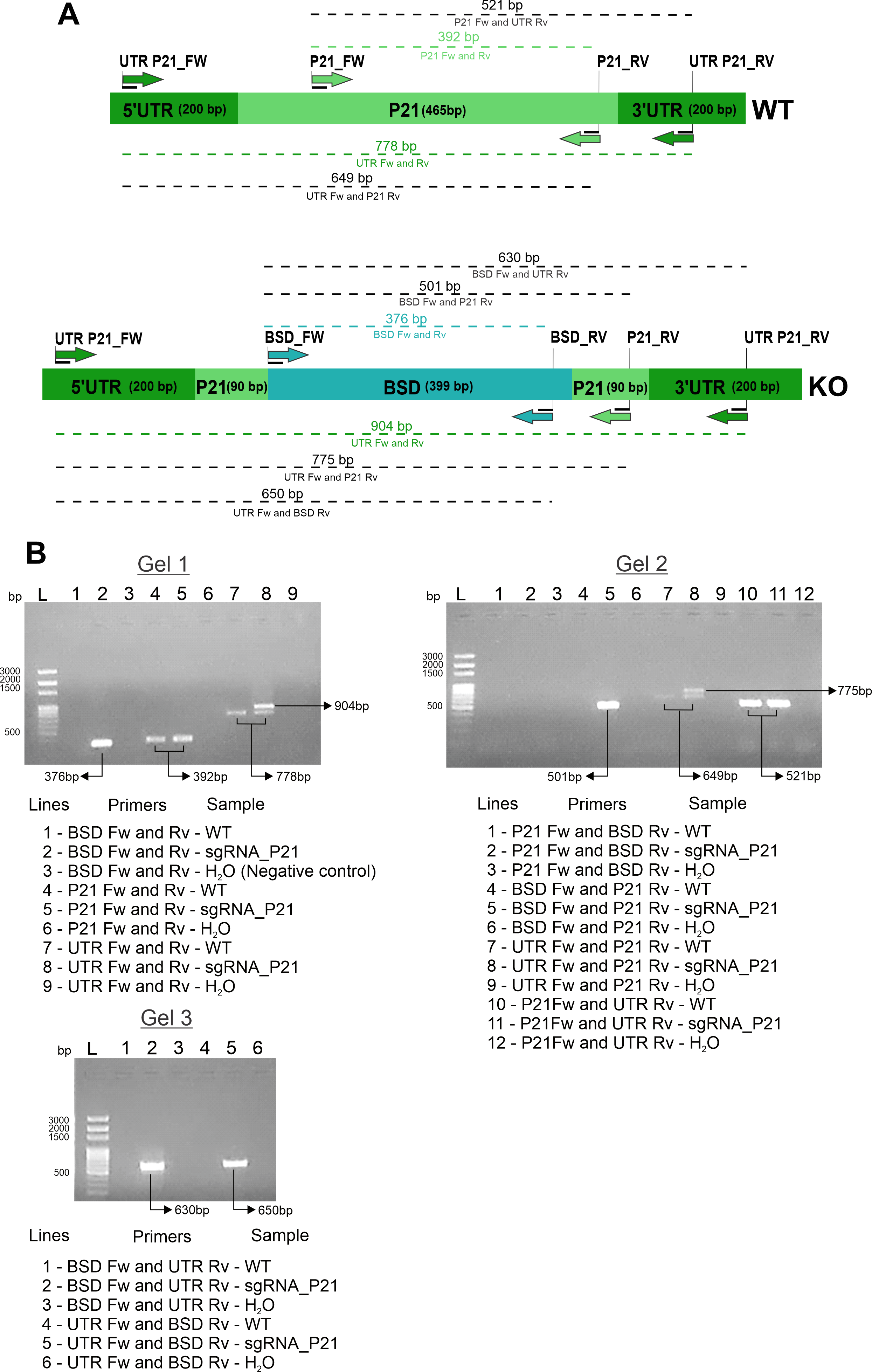

Supplement: Supplementary Figure 2 — PCR profile of amplicons obtained from a mixed population. (A) Schematic representation of the P21 gene locus in WT and knockout clones. All primer combinations used as well as amplicon sizes are shown in the figure. (B) Agarose gels show amplicons obtained by PCR of the WT and sgRNA_P21 populations using different sets of primers. The results indicated a mixed population, with hemi- or double-knockout parasites prior to cloning by limiting dilution. The schematic representation in Figure 2 shows the sites of primer annealing, and Table S2 lists all possible amplicons that can be obtained with the different primer combinations. WT, wild type; sgRNA_P21, parasites transfected with sgRNA and DNA donor before cloning (mixed population). [file Image_2.tif]

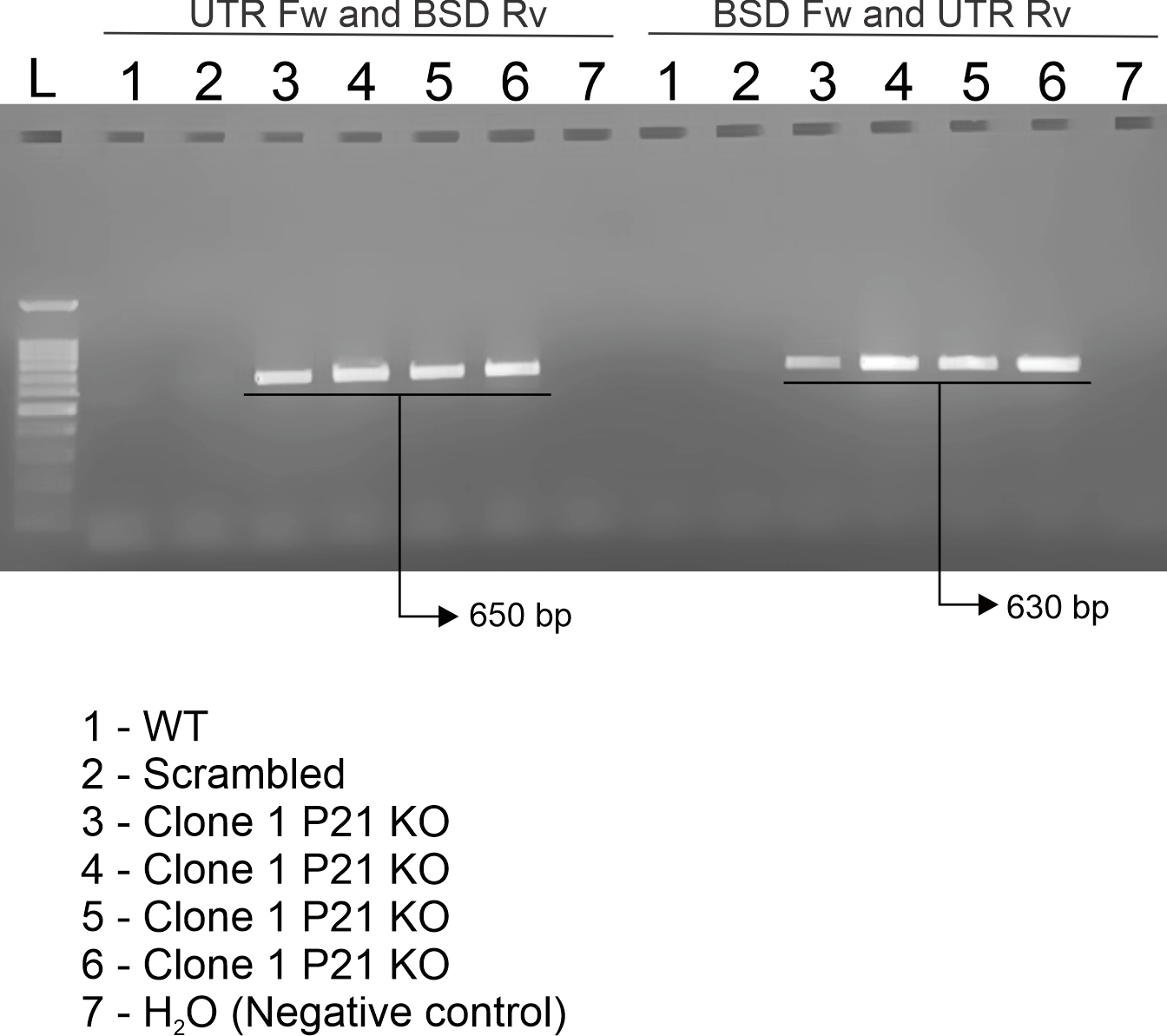

Supplement: Supplementary Figure 3 — Confirmation of insertion of the Bsd gene within the P21 UTR in knockout clones. Agarose gel shows amplicons obtained by PCR of the WT, Scrambled and 4 knockout clones using UTR Fw/BSD Rv and BSD Fw/UTR Rv primers combination. [file Image_3.tif]

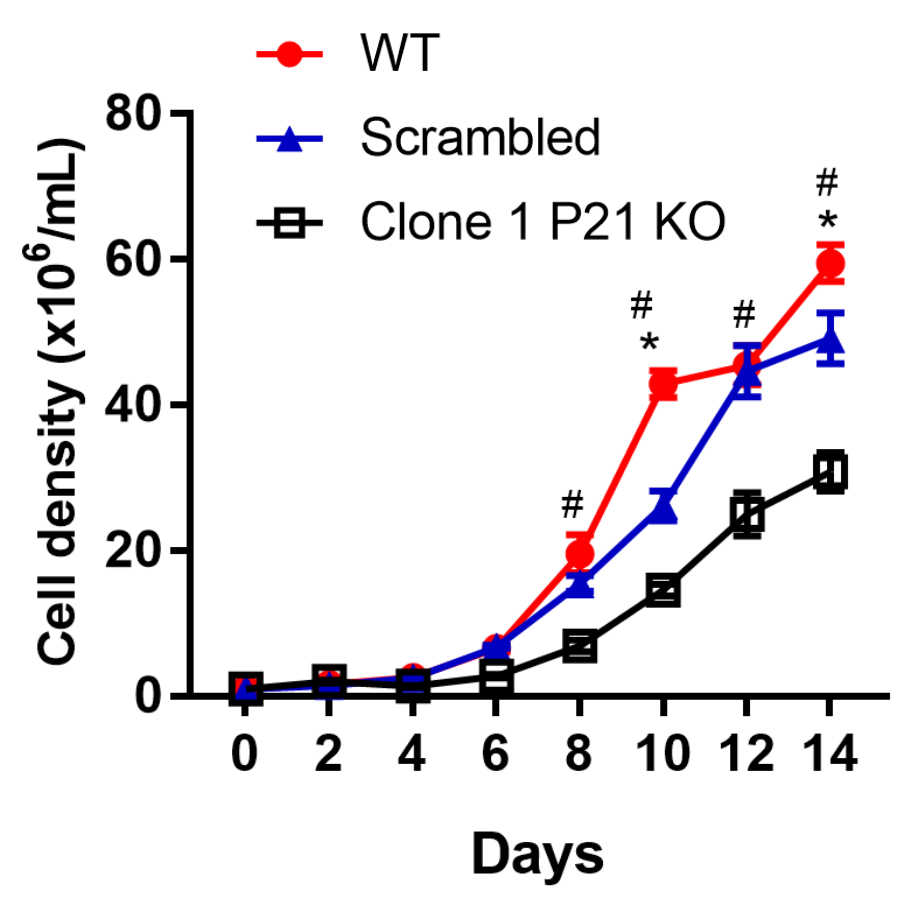

Supplement: Supplementary Figure 4 — Growth curve of epimastigotes comparing WT, Scrambled and Clone 1 P21 KO groups. Epimastigote growth curve for 14 days comparing WT, Scrambled and Clone 1 P21 KO parasites. The graph shows the representative mean ± SD of one experiment performed in sextuplicate. The comparison was performed by two-way ANOVA and Sidak’s test for multiple comparisons. *indicated a statistical difference between WT and Scrambled (p < 0,05). #indicated a statistical difference between WT, Scrambled and Clone 1 P21 KO. [file Image_4.tif]

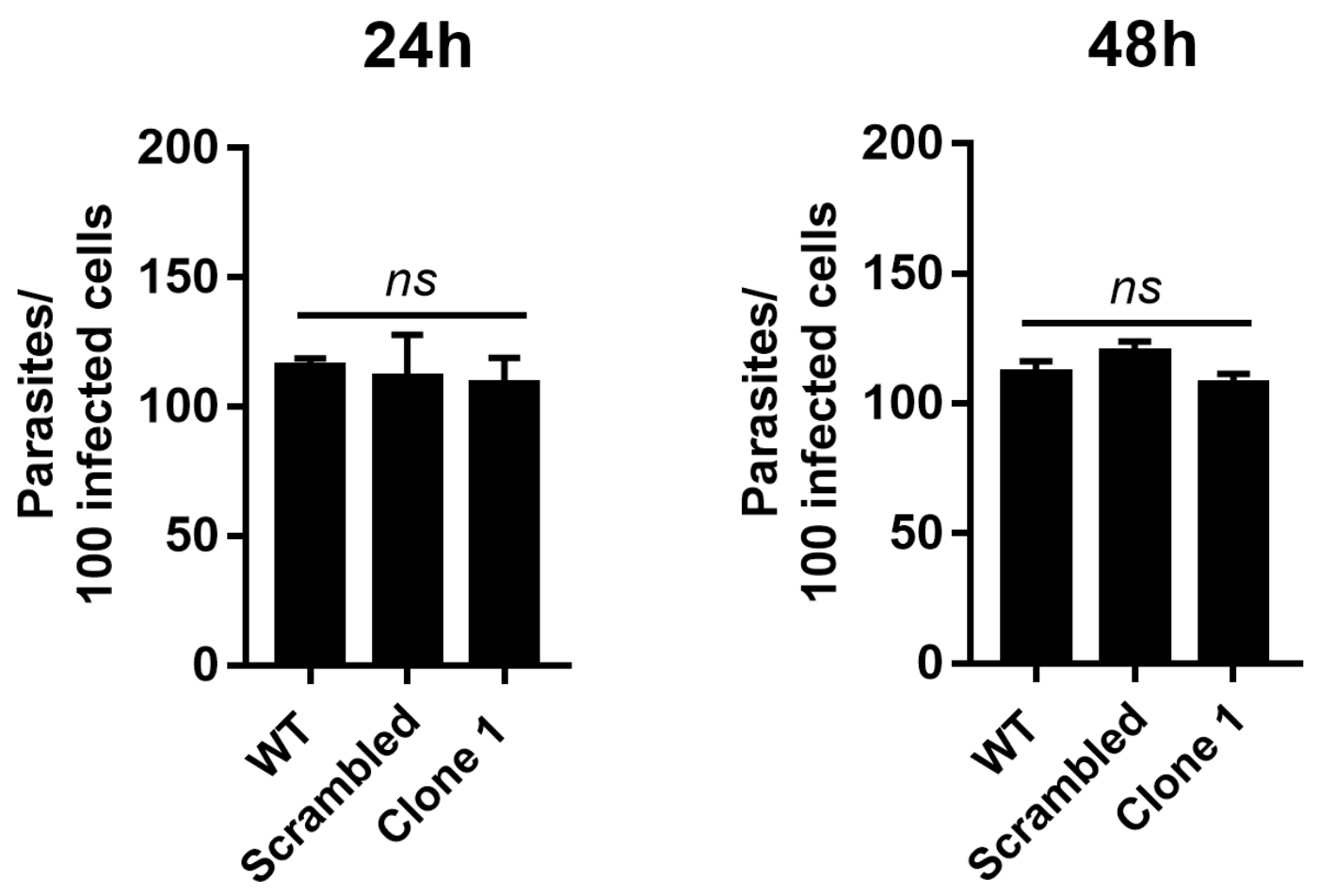

Supplement: Supplementary Figure 5 — Replication of intracellular amastigotes in HeLa cells after 24 and 48 hours of invasion. Total number of parasites in 100 infected cells. The graph shows the representative mean ± SD of one experiment performed in quintuplicate. The comparison was performed by Kruskal-Wallis test and Dunn’s teste for multiple comparison. ns, no significant differences. [file Image_5.tif]
