# Supplementary material for: Ablation of the P21 Gene of Trypanosoma cruzi Provides Evidence of P21 as a Mediator in the Control of Epimastigote and Intracellular Amastigote Replication
Source: Front Cell Infect Microbiol. 2022 Feb 18;12:799668. doi: 10.3389/fcimb.2022.799668 (PMC8895596; doi:10.3389/fcimb.2022.799668)
Supplement: Supplementary file 6 [file Table_1.docx]

**Supplementary Material**

**sgRNA sequence**

Legend

**Start and stop codon**

sgRNA sequence

**PAM**

Homology arms

>TcYC6_0032830 | Trypanosoma cruzi YC6 | lenght=465

**ATG**CGGTTTGTTTTTTTTCTTCTCGTCCTTTTTCTCGCCTGCAGCGTGTCGGCCGAGGAGGTGGTGAATCGGGGATACAACCACAAGGAGCCCCATAAACGCCACCATCAATCTTTTGGACGCCATCGTCATGTGCGCAGGGAAGAAATGAGGAATGCGACGGGGGTGGGATGCAGAGGAGAGATAAGTCGGTACTGCCAAACCCCCGTCACCGGCTTCTACGAGTACTGGTGGAAGTGTCTTTCCGACAATATGGACCGGTTCAGCACGCCCGACTGCCAGACGTACATAAATGGCATGATTGCCTGC**AGG**AACTTTACCGTTTCTTCCTACGGTCCAGGGGAGCAGTCTCCAGACGGGTTGGTGAAGCACCTGCTTCATTCAGAAAACGAGTCGATCCCCAACGAATGCAGGAACTCCAGATTCTACAAAGATGCCGTGGTTGGATTCCACAGACGCCAG**TAA**

>TcYC6_0032830 | Trypanosoma cruzi Y C6 | P21 | genomic | TcYC6_Chr17 reverse | (geneCodeStart-200 to geneCodeEnd+200)| length=865

AATACGTATGAATGTCTCCATCCACATGTCATGTGGAGTTTTAGCGTCAAACTTCTGTGG

GTTTATGTTTTAAATTTTTTTTTTTCTTTTTTCTTCTTTCCCACCACCACCACGGCCGTG

TGAGAATAGGCTTTGTAAAAAGAATTTAATTTTACGGACACATCTCGCTAAACAGCAGCA

ACAGCAGCAGCAGGAGGAGC**ATG**CGGTTTGTTTTTTTTCTTCTCGTCCTTTTTCTCGCCT

GCAGCGTGTCGGCCGAGGAGGTGGTGAATCGGGGATACAACCACAAGGAGCCCCATAAAC

GCCACCATCAATCTTTTGGACGCCATCGTCATGTGCGCAGGGAAGAAATGAGGAATGCGA

CGGGGGTGGGATGCAGAGGAGAGATAAGTCGGTACTGCCAAACCCCCGTCACCGGCTTCT

ACGAGTACTGGTGGAAGTGTCTTTCCGACAATATGGACCGGTTCAGCACGCCCGACTGCC

AGACGTACATAAATGGCATGATTGCCTGC**AGG**AACTTTACCGTTTCTTCCTACGGTCCAG

GGGAGCAGTCTCCAGACGGGTTGGTGAAGCACCTGCTTCATTCAGAAAACGAGTCGATCC

CCAACGAATGCAGGAACTCCAGATTCTACAAAGATGCCGTGGTTGGATTCCACAGACGCC

AG**TAA**CGCCTCGGAATGGGGGAATTCTTCACGGCTGCAGGAGAGGAGGAGAGAAGGCGGC

CTGACAACTGTATGGAAAAATGAACCACAACCCTCCTCCTCCTCCTCCTGCAAGACTTAA

TTGGACGTTGGGCTGCTGGAAAGAGGCAGGAGGAAAAGACACACTGCATTCCTTTAGAAA

TACAAATAAGGACTACTACTCTTTT

**DNA Donor sequence**

Legend

Homology arms

**Start e stop codon of P21**

Bsd sequence

UTR sequence

CAGCAGGAGGAGC**ATG**CGGTTTGTTTTTGTTCTTCTCGTCCTTTTTCTCGCCTGCAGCGTGTCGGCCGTGGAGGTGATGAAGCGGGGATACAACCACAAGGAGATGGCCAAGCCTTTGTCTCAAGAAGAATCCACCCTCATTGAAAGAGCAACGGCTACAATCAACAGCATCCCCATCTCTGAAGACTACAGCGTCGCCAGCGCAGCTCTCTCTAGCGACGGCCGCATCTTCACTGGTGTCAATGTATATCATTTTACTGGGGGACCTTGTGCAGAACTCGTGGTGCTGGGCACTGCTGCTGCTGCGGCAGCTGGCAACCTGACTTGTATCGTCGCGATCGGAAATGAGAACAGGGGCATCTTGAGCCCCTGCGGACGGTGCCGACAGGTGCTTCTCGATCTGCATCCTGGGATCAAAGCCATAGTGAAGGACAGTGATGGACAGCCGACGGCAGTTGGGATTCGTGAATTGCTGCCCTCTGGTTATGTGTGGGAGGGCTAAGTGAAGCACCTGCTTCATTCAGAAAAAGAGTCGATCCCCAGCGAATGCAGGAACTCCAGATTCTACAAAGATACCGTGGTCGGATTCCACAGACGCCAG**TAA**
