# Supplementary material for: Ablation of the P21 Gene of Trypanosoma cruzi Provides Evidence of P21 as a Mediator in the Control of Epimastigote and Intracellular Amastigote Replication
Source: Front Cell Infect Microbiol. 2022 Feb 18;12:799668. doi: 10.3389/fcimb.2022.799668 (PMC8895596; doi:10.3389/fcimb.2022.799668)
Supplement: Supplementary file 7 [file Table_2.docx]

| Primers | | |  |
| --- | --- | --- | --- |
| 1 | TcP21_sgRNA_BamHI Fw | GATCGGATCC***TAAATGGCATGATTGCCTGC***GTTTTAGAGCTAGAAATAGC | |
| 2 | Scrambled_BamHI Fw | GATCGGATCC***GCACTACCAGAGCTAACTCA***GTTTTAGAGCTAGAAATAGC | |
| 3 | sgRNA_BamHI_ Rv all genes | CAGTGGATCCAAAAAAGCACCGACTCGGTG | |
| 4 | Tcp21 ultramer Fw | CAGCAGGAGGAGCCTGCGGTTTGTTTTTGTTCTTCTCGTCCTTTTTCTCG CCTGCAGCGTGTCGGCCGTGGAGGTGGTGAATCGGGGATACAACCACAAG GAGATGGCCAAGCCTTTGTC TCAAG | |
| 5 | Tcp21_Ultramer Rv | TTACTGGCGTCTGTGGAATCCGACCACGGCATCTTTGTAGAATCTGGAGT TCCTGCATTCGTTGGGGATCGACTCTTTTTCTGAATGAAGCAGGTGCTTC ATTAGCCCTCCCACACATAACC | |
| 6 | P21 Fw | GATACAACCACAAGGAGCC | |
| 7 | P21 Rv | TTACTGGCGTCTGTGGAATC | |
| 8 | UTR P21 Fw | GCCTCCATCCACATTTCATG | |
| 9 | UTR P21 Rv | AACGTCCAATTAGGTCTTGTA | |
| 10 | BSD Fw | ATGGCCAAGCCTTTGTCTCA | |
| 11 | BSD Rv | AGGGCAGCAATTCACGAATC | |
| 12 | TcHGPRT Fw | CTACAAGGGAAAGGGTCTGC | |
| 13 | TcHGPRT Rv | ACCGTAGCCAATCACAAAGG | |
| 14 | TcMVK Fw | CGGCCGCGACATTTGGT | |
| 15 | TcMVK Rv | GGCACTTCTAGGGCACGCAG | |

**Table S1**. Primers used to generate CRISPR/Cas9 constructs for *T. cruzi*, knockout confirmation and RT-PCR.
