# Supplementary material for: Ablation of the P21 Gene of Trypanosoma cruzi Provides Evidence of P21 as a Mediator in the Control of Epimastigote and Intracellular Amastigote Replication
Source: Front Cell Infect Microbiol. 2022 Feb 18;12:799668. doi: 10.3389/fcimb.2022.799668 (PMC8895596; doi:10.3389/fcimb.2022.799668)
Supplement: Supplementary file 8 [file Table_3.docx]

**Table S2.** Expected amplicon sizes expected in the WT and *P21* knockout populations.

| Primers | Populations | Amplicon size |
| --- | --- | --- |
| P21 Fw and Rv | WT | 392 bp |
|  | P21 KO | No amplification |
| UTR Fw and Rv | WT | 778 bp |
|  | P21 KO | 904 bp |
| BSD Fw and Rv | WT | No amplification |
|  | P21 KO | 376 bp |
| UTR Fw and BSD Rv | WT | No amplification |
|  | P21 KO | 650 bp |
| BSD Fw and UTR Rv | WT | No amplification |
|  | P21 KO | 630 bp |
| P21 Fw and BSD Rv | WT | No amplification |
|  | P21 KO | No amplification |
| BSD Fw and P21 Rv | WT | No amplification |
|  | P21 KO | 501 bp |
| UTR Fw and P21 Rv | WT | 649 bp |
|  | P21 KO | 775 bp |
| P21 Fw and UTR Rv | WT | 521 bp |
|  | P21 KO | No amplification |
