# Supplementary material for: Ablation of the P21 Gene of Trypanosoma cruzi Provides Evidence of P21 as a Mediator in the Control of Epimastigote and Intracellular Amastigote Replication
Source: Front Cell Infect Microbiol. 2022 Feb 18;12:799668. doi: 10.3389/fcimb.2022.799668 (PMC8895596; doi:10.3389/fcimb.2022.799668)
Supplement: Supplementary file 9 [file Table_4.docx]

**Supplementary material_sequencing**

**Sequencing of *P21* knockout clones**

Schematic gene *loci* (*P21* knockout) and its adjacent UTR regions.


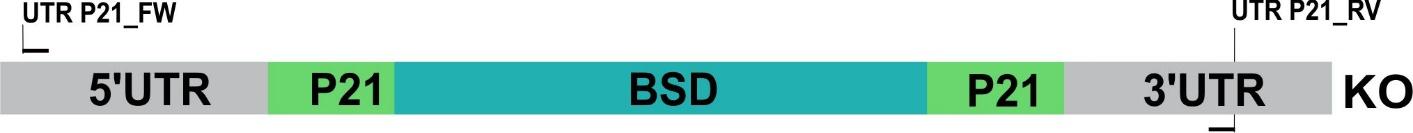


Query:

ACAAATACGTATGAATGCCTCCATCCACATTTCATGTGGAGTTTTAGCGTCAAACTTCTGTGGGTTTATTTTTAATCTTTTTTTTTAATCTACTTTTCCAACTCACGCTGCACCACGGCCGTGTGAGAATAGGCTTTGTAAAAGGAATTTAATTTTACGGACACATCTCGCTAAACAGCAGCAACAACAGCAGGAGGAGCATGCGGTTTGTTTTTGTTCTTCTCGTCCTTTTTCTCGCCTGCAGCGTGTCGGCCGTGGAGGTGATGAAGCGGGGATACAACCACAAGGAGATGGCCAAGCCTTTGTCTCAAGAAGAATCCACCCTCATTGAAAGAGCAACGGCTACAATCAACAGCATCCCCATCTCTGAAGACTACAGCGTCGCCAGCGCAGCTCTCTCTAGCGACGGCCGCATCTTCACTGGTGTCAATGTATATCATTTTACTGGGGGACCTTGTGCAGAACTCGTGGTGCTGGGCACTGCTGCTGCTGCGGCAGCTGGCAACCTGACTTGTATCGTCGCGATCGGAAATGAGAACAGGGGCATCTTGAGCCCCTGCGGACGGTGCCGACAGGTGCTTCTCGATCTGCATCCTGGGATCAAAGCCATAGTGAAGGACAGTGATGGACAGCCGACGGCAGTTGGGATTCGTGAATTGCTGCCCTCTGGTTATGTGTGGGAGGGCTAAGTGAAGCACCTGCTTCATTCAGAAAAAGAGTCGATCCCCAGCGAATGCAGGAACTCCAGATTCTACAAAGATACCGTGGTCGGATTCCACAGACGCCAGTAACACCTCGGAATGGGGGGATTCTTCACGGCTGCAGGAGAGGAGGAGAGAAGGGGCAGCCTGACAACTGTATGGAAAAATGAACCACTACCCTCTTCCTCCTCTTCCTCCTACAAGACCTAATTGGACGTTGGGCTGCTGGAGAGAGGCAGGAGGAAAAGACACACTGCGTTCCTCTACAAATAAAAATAAGGACTACTCCT

5’UTR: 1 - 200 bp

P21_1: 201 - 290 bp

BSD: 291 - 689 bp

P21_2: 690 - 791 bp

3’UTR: 792 - 991 bp

**Sequencing Results**

**WT_UTR_Fw:**

TRKRRKYRTAYWKAWRGATTATRTTTTAATTTTTTTTTTTCTTWTTTCTTYCCCACCMCCMCCMCGGCCSKGKGARAATAGGCTTTGTAAAAARAATTTAWTTTTWCGGACACWTYTYSYTAAACAGCAGCAACAGCAGCAACAGGAGGAGCATGSGGTTKGTTTTTTTTYTTCTCGTCCTTTTTYTCSCCKGCAGCGKGTCGGCCGAGGAGGKGGKGAAGCGGGGATACAACCMCAAGGAGCCCCATAAACSCCMCCATCAATYTTTTGGACSCCATCGTCATGKGSGCAGGGAARAAATGAGGAAKGCGACGGGGGKGGGATGCARAGRARARATAAKTCGGTACTGCCAAACCCCCGYCACCGGYTTCTACRAGWACKGGKGGARGKGTYTTTCCRAAAATATGGACSGGTTCAGCACGCCCGACTGCCRRACKTACATAAATGGCATGATKGCCKGCAGGAACTTTACCGTTTCTTCCTACGGYCCAGGGGAGCAGYCTCCAGACGGGTKGGTGAAGCACCTGCTTCWTTCARAAAACRAGTCGATCCCCAACRAAKGCAGGAACTCCARATTCTACAAGGATGCCGKGGTKGGATTCCACARACGCCAGTAACGCCTCGGAATGGGGGGATTCTTCACGGCTGCAGGAGAGGAGGAGAGAAGGCGGCCTGACAACTGTATGGAAAAATRAACCACAATCCTCCTCCTCCTCCTACAASACATTTKKGRMGKTWWAAAATA


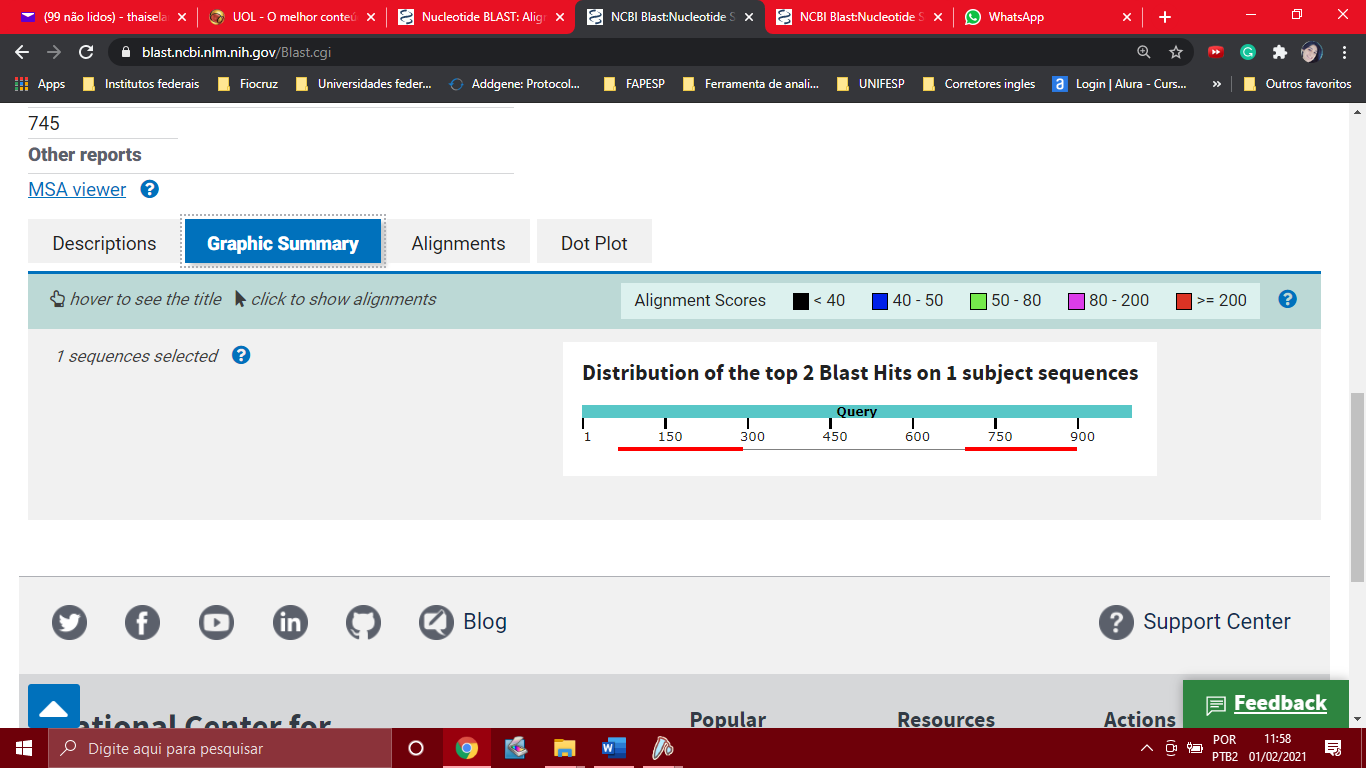
**Blast Query x WT_UTR_Fw:**

Range 1: 516 to 716

|  | | | | | |
| --- | --- | --- | --- | --- | --- |
| Score | Expect | Identities | Gaps | Strand |  |
| 274 bits(148) | 2e-77 | 182/203(90%) | 2/203(0%) | Plus/Plus |  |

Query 690 GTGAAGCACCTGCTTCATTCAGAAAAAGAGTCGATCCCCAGCGAATGCAGGAACTCCAGA 749

|||||||||||||||| |||| |||| |||||||||||| | || |||||||||||| |

Sbjct 516 GTGAAGCACCTGCTTCWTTCARAAAACRAGTCGATCCCCAACRAAKGCAGGAACTCCARA 575

Query 750 TTCTACAAAGATACCGTGGTCGGATTCCACAGACGCCAGTAACACCTCGGAATGGGGGGA 809

|||||||| ||| ||| ||| |||||||||| ||||||||||| ||||||||||||||||

Sbjct 576 TTCTACAAGGATGCCGKGGTKGGATTCCACARACGCCAGTAACGCCTCGGAATGGGGGGA 635

Query 810 TTCTTCACGGCTGCAGGAGAGGAGGAGAGAAGGGGCAGCCTGACAACTGTATGGAAAAAT 869

||||||||||||||||||||||||||||||||| | |||||||||||||||||||||||

Sbjct 636 TTCTTCACGGCTGCAGGAGAGGAGGAGAGAAGGCG--GCCTGACAACTGTATGGAAAAAT 693

Query 870 GAACCACTACCCTCTTCCTCCTC 892

|||||| | |||| ||||||||

Sbjct 694 RAACCACAATCCTCCTCCTCCTC 716

Range 2: 19 to 242

| **Alignment statistics for match #2** | | | | | |
| --- | --- | --- | --- | --- | --- |
| Score | Expect | | Identities | Gaps | Strand |
| 206 bits(111) | 7e-57 | 183/229(80%) | | 9/229(3%) | Plus/Plus |

Query 66 TTAT-TTTTAATCtttttttttAATCTACTTTTCCAACTCACGCTGCACCACGGCCGTGT 124

|||| ||||||| ||||||||| ||| |||| | ||| | | || ||||| |

Sbjct 19 TTATRTTTTAAT-TTTTTTTTT--TCT-TWTTTCTTYCCCAC-CMCCMCCMCGGCCSKGK 73

Query 125 GAGAATAGGCTTTGTAAAAGGAATTTAATTTTACGGACACATCTCGCTAAACAGCAGCAA 184

|| |||||||||||||||| |||||| |||| ||||||| | | |||||||||||||

Sbjct 74 GARAATAGGCTTTGTAAAAARAATTTAWTTTTWCGGACACWTYTYSYTAAACAGCAGCAA 133

Query 185 CA--A-CAGCAGGAGGAGCATGCGGTTTGTTTTTGTTCTTCTCGTCCTTTTTCTCGCCTG 241

|| | || ||||||||||||| |||| |||||| || |||||||||||||| || || |

Sbjct 134 CAGCAGCAACAGGAGGAGCATGSGGTTKGTTTTTTTTYTTCTCGTCCTTTTTYTCSCCKG 193

Query 242 CAGCGTGTCGGCCGTGGAGGTGATGAAGCGGGGATACAACCACAAGGAG 290

||||| |||||||| ||||| | ||||||||||||||||| |||||||

Sbjct 194 CAGCGKGTCGGCCGAGGAGGKGGKGAAGCGGGGATACAACCMCAAGGAG 242

**Scrambled_UTR_Fw:**

GRKTCASTTMTGTGGRTTTATRTTTTAATTTTTTTTTTTCTTWTTTCTTTCCCACCMCCMCCACGGCCSYGKGARAATAGGCTTTGTAAAAARAATTTAWTTTTWCGGACACWTCTCSYTAAACAGCAGCAACAGCAGCAACAGGRGGAGCAKGSGGTTKGTTTTTTTTYTTCTCGTCCTTTTTYTCSCCKGCAGCGKGTCGGCCGAGGAGGKGGKGAAGCGGGGATACAACCMCAAGGAGCCCCATAAACSCCMCCATCAWTYTTTTGGASSCCAYCGTCATGKGSGCAGGGAARAAATGAGGAAKGCGACGGGGGKGGGAKGCARAGRARARATAAKTCGGTAYTGCCAAACCCCCGYCACCGGYTTYTACRAGTACKGGKGGARGKGTYTTTCCRAAAATATGGACCGGTTCAGCACGCCCGACTGCCRRACKTACATAAATGGCRKGATKGCCTGCAGGAACTTTACCGTTTCTTCYTACGGYCCAGGGGAGCAGTCTCCARACGGGTTGGTGAAGMACCTGCTTCWTTCARAAAACRAGTCGATCCCCAACRAATGCAGGAACTCCARATTCTACAAGGATGCCGKGGTKGGATTCCACARACGCCAGTAACGCCTCGGAATGGGGGGATTCTTCACGGCTGCAGGAGAGGAGGAGAGAAGGCGGCCTGACAACTGTATGGAAAAATGAACCACAATCCTCCTCCTCCTCCTACAAGACATTKKKKGGRGKKWWWAAA

**Blast Query x Scrambled_UTR_Fw:**


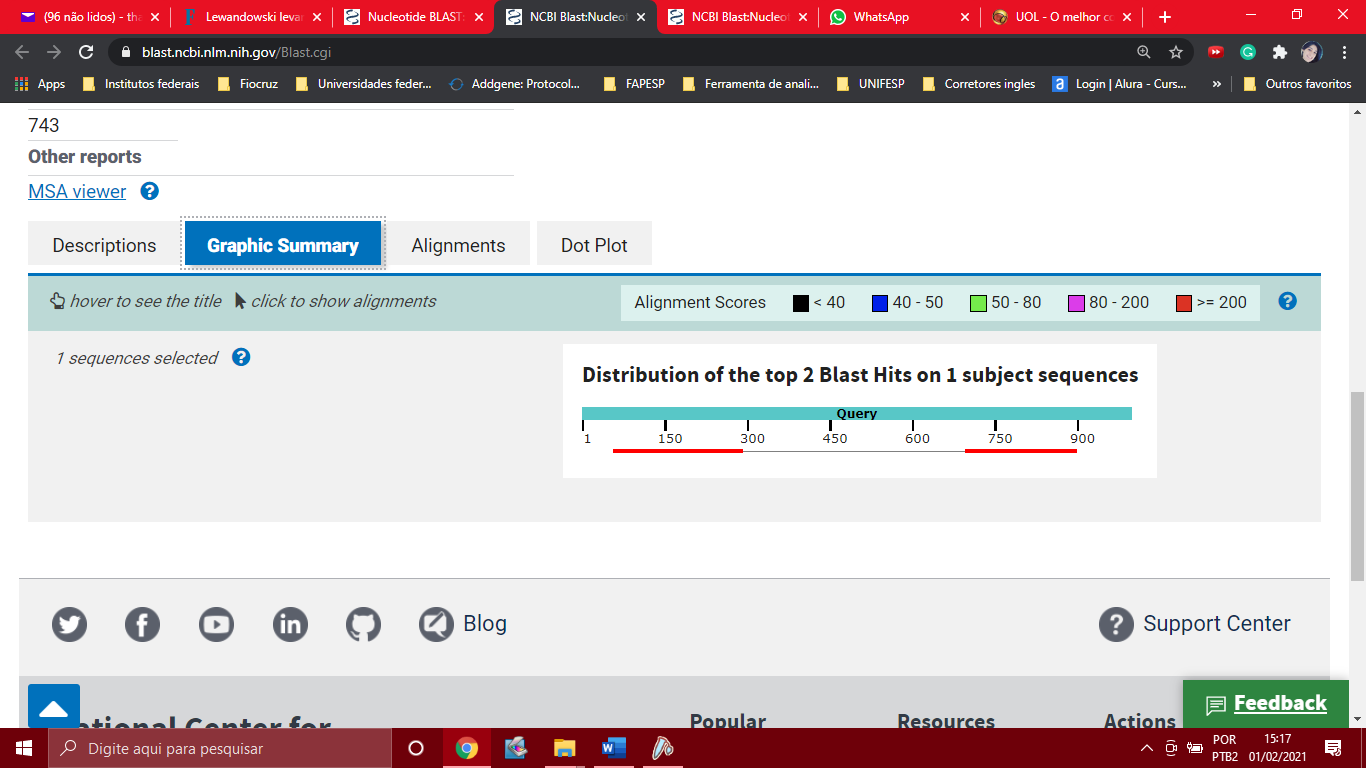


Range 1: 515 to 715

|  | | | | |
| --- | --- | --- | --- | --- |
| Score | Expect | Identities | Gaps | Strand |
| 278 bits(150) | 1e-78 | 183/203(90%) | 2/203(0%) | Plus/Plus |

Query 690 GTGAAGCACCTGCTTCATTCAGAAAAAGAGTCGATCCCCAGCGAATGCAGGAACTCCAGA 749

|||||| ||||||||| |||| |||| |||||||||||| | ||||||||||||||| |

Sbjct 515 GTGAAGMACCTGCTTCWTTCARAAAACRAGTCGATCCCCAACRAATGCAGGAACTCCARA 574

Query 750 TTCTACAAAGATACCGTGGTCGGATTCCACAGACGCCAGTAACACCTCGGAATGGGGGGA 809

|||||||| ||| ||| ||| |||||||||| ||||||||||| ||||||||||||||||

Sbjct 575 TTCTACAAGGATGCCGKGGTKGGATTCCACARACGCCAGTAACGCCTCGGAATGGGGGGA 634

Query 810 TTCTTCACGGCTGCAGGAGAGGAGGAGAGAAGGGGCAGCCTGACAACTGTATGGAAAAAT 869

||||||||||||||||||||||||||||||||| | |||||||||||||||||||||||

Sbjct 635 TTCTTCACGGCTGCAGGAGAGGAGGAGAGAAGGCG--GCCTGACAACTGTATGGAAAAAT 692

Query 870 GAACCACTACCCTCTTCCTCCTC 892

||||||| | |||| ||||||||

Sbjct 693 GAACCACAATCCTCCTCCTCCTC 715

Range 2: 8 to 241

|  | | | | |
| --- | --- | --- | --- | --- |
| Score | Expect | Identities | Gaps | Strand |
| 220 bits(119) | 2e-61 | 192/239(80%) | 9/239(3%) | Plus/Plus |

Query 56 TTCTGTGGGTTTAT-TTTTAATCtttttttttAATCTACTTTTCCAACTCACGCTGCACC 114

|| ||||| ||||| ||||||| ||||||||| ||| |||| | ||| | | ||

Sbjct 8 TTMTGTGGRTTTATRTTTTAAT-TTTTTTTTT--TCT-TWTTTCTTTCCCAC-CMCCMCC 62

Query 115 ACGGCCGTGTGAGAATAGGCTTTGTAAAAGGAATTTAATTTTACGGACACATCTCGCTAA 174

|||||| | || |||||||||||||||| |||||| |||| ||||||| |||| |||

Sbjct 63 ACGGCCSYGKGARAATAGGCTTTGTAAAAARAATTTAWTTTTWCGGACACWTCTCSYTAA 122

Query 175 ACAGCAGCAACAACAG---CAGGAGGAGCATGCGGTTTGTTTTTGTTCTTCTCGTCCTTT 231

|||||||||||| ||| |||| |||||| | |||| |||||| || ||||||||||||

Sbjct 123 ACAGCAGCAACAGCAGCAACAGGRGGAGCAKGSGGTTKGTTTTTTTTYTTCTCGTCCTTT 182

Query 232 TTCTCGCCTGCAGCGTGTCGGCCGTGGAGGTGATGAAGCGGGGATACAACCACAAGGAG 290

|| || || |||||| |||||||| ||||| | ||||||||||||||||| |||||||

Sbjct 183 TTYTCSCCKGCAGCGKGTCGGCCGAGGAGGKGGKGAAGCGGGGATACAACCMCAAGGAG 241

**P2B10_UTR_Rv:**

TSKCWTKKCYTGRWTWWWTGCWCTGYWGTSAGGCYGCCTTCWCTCCTCCTCTCCTGCAGCCGTGAAGMATCCYCCCATTCCGAGGCGTTACTGGCGTCTGTGGAATCCAACCACGGCATCCTTGTAGAATCTGGAGTTCCTGCMTTCGTTGGGGATCGACTCTTTTTCTGAATGAAGCAGGTGCTTCMTTAGCCCTCCCACACATAACCAGAGGGCAGCAATTCACGAATCCCAACTGCCGKCGGCTGTCCATCACTGTCCTTCACTATGGCTTTGATCCCAGGATGCAGATCGAGAAGCACCTGTCGGCACCGTCCGCAGGGGCTCAAGATGCCCCTGTTCTCATTTCCKATCGCGACGATACAAGTCAGGTTGCCAGCTGCCGCAGCAGCAGCAGTGCCCAGCACCACGAGTTCTGCACAAGGTCCCYCWKTWAAATGATATACATTGACACCAGTGAAGATGCGGCCGTCGCTAGAGAGAGCTGCGCTGGCGACGCTGTAGTCTTCAGAGATGGGGATGCTGTTGATTGTAGCCGTTGCTCTTTCAATGAGGGTGGATTCTTCTTGAGACAAAGGCTTGGYCATCTCCTTGKGKTKKATYCCCSRWTCMMCAMCTCYCMCSGTCGASCACMGSYTGCWGCGAGARAAAGGACGAGAGARAAMAAACAAMACMGCATGCTGCTCCTSTGCTGCTGCTGCTGCTGCTKTAKCRAGATGWGTCGTYAAATAAAATACTCTTTAYAAARCYATACTCAYACGCGYGTGTGTTGAAGAGAGGAGAACTTACWAGCCGAGTTGSCTACTCCTGATGTGTGGGGCASAA

**Reverso Complementar_P2B10_UTR_Rv**

TTSTGCCCCACACATCAGGAGTAGSCAACTCGGCTWGTAAGTTCTCCTCTCTTCAACACA

CRCGCGTRTGAGTATRGYTTTRTAAAGAGTATTTTATTTRACGACWCATCTYGMTAMAGC

AGCAGCAGCAGCAGCASAGGAGCAGCATGCKGTKTTGTTTKTTYTCTCTCGTCCTTTYTC

TCGCWGCARSCKGTGSTCGACSGKGRGAGKTGKKGAWYSGGGRATMMAMCMCAAGGAGAT

GRCCAAGCCTTTGTCTCAAGAAGAATCCACCCTCATTGAAAGAGCAACGGCTACAATCAA

CAGCATCCCCATCTCTGAAGACTACAGCGTCGCCAGCGCAGCTCTCTCTAGCGACGGCCG

CATCTTCACTGGTGTCAATGTATATCATTTWAMWGRGGGACCTTGTGCAGAACTCGTGGT

GCTGGGCACTGCTGCTGCTGCGGCAGCTGGCAACCTGACTTGTATCGTCGCGATMGGAAA

TGAGAACAGGGGCATCTTGAGCCCCTGCGGACGGTGCCGACAGGTGCTTCTCGATCTGCA

TCCTGGGATCAAAGCCATAGTGAAGGACAGTGATGGACAGCCGMCGGCAGTTGGGATTCG

TGAATTGCTGCCCTCTGGTTATGTGTGGGAGGGCTAAKGAAGCACCTGCTTCATTCAGAA

AAAGAGTCGATCCCCAACGAAKGCAGGAACTCCAGATTCTACAAGGATGCCGTGGTTGGA

TTCCACAGACGCCAGTAACGCCTCGGAATGGGRGGATKCTTCACGGCTGCAGGAGAGGAG

GAGWGAAGGCRGCCTSACWRCAGWGCAWWWAWYCARGMMAWGMSA

**Blast query x P2B10_UTR_Rv:**


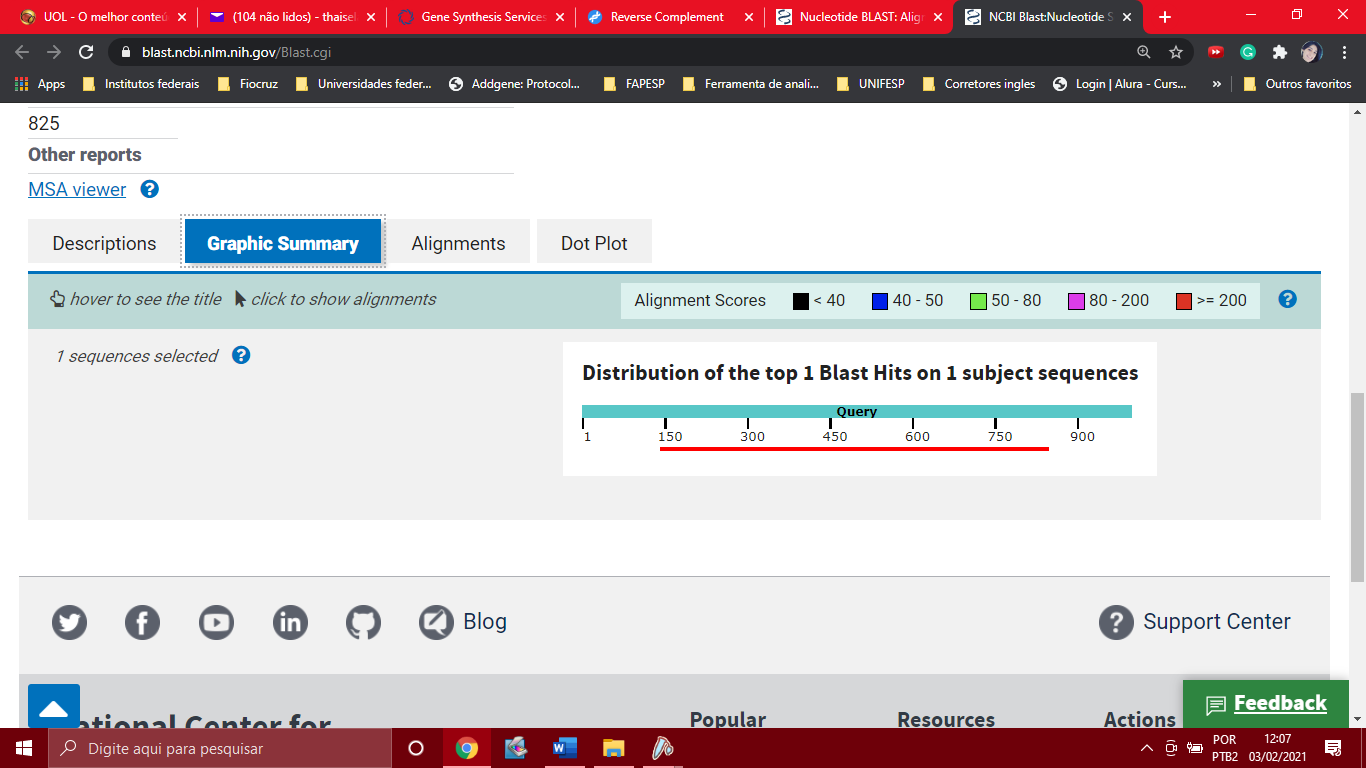


| Range 1: 84 to 789 | | | | |
| --- | --- | --- | --- | --- |
| Score | Expect | Identities | Gaps | Strand |
| 1016 bits(550) | 0.0 | 649/711(91%) | 14/711(1%) | Plus/Plus |

Query 141 AAAG-GAATTTAATTTTACGGACACATCTCGCTA-AACAGCAGCAACAACAGC--AGGAG 196

|||| | |||| |||| || ||| ||||| | || | |||||||| || |||| |||||

Sbjct 84 AAAGAGTATTTTATTTRAC-GACWCATCTYGMTAMAGCAGCAGCAGCAGCAGCASAGGAG 142

Query 197 GAGCATGCGGT-TTGTTTTTGTTCTTCTCGTCCTTTTTCTCGCCTGCA-GC-GTG-TCGG 252

||||||| || |||||| | || ||||||||||| |||||| ||| | ||| |||

Sbjct 143 CAGCATGCKGTKTTGTTTKTTYTC-TCTCGTCCTTTYTCTCGC-WGCARSCKGTGSTCGA 200

Query 253 CCGTG-GAGGTGATGAAGCGGGGATACAACCACAAGGAGATGGCCAAGCCTTTGTCTCAA 311

| | | ||| || || ||| || | | |||||||||| |||||||||||||||||

Sbjct 201 CSGKGRGAGKTGKKGAWYSGGGRAT-MMAMCMCAAGGAGATGRCCAAGCCTTTGTCTCAA 259

Query 312 GAAGAATCCACCCTCATTGAAAGAGCAACGGCTACAATCAACAGCATCCCCATCTCTGAA 371

||||||||||||||||||||||||||||||||||||||||||||||||||||||||||||

Sbjct 260 GAAGAATCCACCCTCATTGAAAGAGCAACGGCTACAATCAACAGCATCCCCATCTCTGAA 319

Query 372 GACTACAGCGTCGCCAGCGCAGCTCTCTCTAGCGACGGCCGCATCTTCACTGGTGTCAAT 431

||||||||||||||||||||||||||||||||||||||||||||||||||||||||||||

Sbjct 320 GACTACAGCGTCGCCAGCGCAGCTCTCTCTAGCGACGGCCGCATCTTCACTGGTGTCAAT 379

Query 432 GTATATCATTTTACTGGGGGACCTTGTGCAGAACTCGTGGTGCTGGGCACTGCTGCTGCT 491

||||||||||| | | |||||||||||||||||||||||||||||||||||||||||||

Sbjct 380 GTATATCATTTWAMWGRGGGACCTTGTGCAGAACTCGTGGTGCTGGGCACTGCTGCTGCT 439

Query 492 GCGGCAGCTGGCAACCTGACTTGTATCGTCGCGATCGGAAATGAGAACAGGGGCATCTTG 551

||||||||||||||||||||||||||||||||||| ||||||||||||||||||||||||

Sbjct 440 GCGGCAGCTGGCAACCTGACTTGTATCGTCGCGATMGGAAATGAGAACAGGGGCATCTTG 499

Query 552 AGCCCCTGCGGACGGTGCCGACAGGTGCTTCTCGATCTGCATCCTGGGATCAAAGCCATA 611

||||||||||||||||||||||||||||||||||||||||||||||||||||||||||||

Sbjct 500 AGCCCCTGCGGACGGTGCCGACAGGTGCTTCTCGATCTGCATCCTGGGATCAAAGCCATA 559

Query 612 GTGAAGGACAGTGATGGACAGCCGACGGCAGTTGGGATTCGTGAATTGCTGCCCTCTGGT 671

|||||||||||||||||||||||| |||||||||||||||||||||||||||||||||||

Sbjct 560 GTGAAGGACAGTGATGGACAGCCGMCGGCAGTTGGGATTCGTGAATTGCTGCCCTCTGGT 619

Query 672 TATGTGTGGGAGGGCTAAGTGAAGCACCTGCTTCATTCAGAAAAAGAGTCGATCCCCAGC 731

|||||||||||||||||| |||||||||||||||||||||||||||||||||||||| |

Sbjct 620 TATGTGTGGGAGGGCTAAK-GAAGCACCTGCTTCATTCAGAAAAAGAGTCGATCCCCAAC 678

Query 732 GAATGCAGGAACTCCAGATTCTACAAAGATACCGTGGTCGGATTCCACAGACGCCAGTAA 791

||| |||||||||||||||||||||| ||| ||||||| |||||||||||||||||||||

Sbjct 679 GAAKGCAGGAACTCCAGATTCTACAAGGATGCCGTGGTTGGATTCCACAGACGCCAGTAA 738

Query 792 CACCTCGGAATGGGGGGATTCTTCACGGCTGCAGGAGAGGAGGAGAGAAGG 842

| |||||||||||| |||| ||||||||||||||||||||||||| |||||

Sbjct 739 CGCCTCGGAATGGGRGGATKCTTCACGGCTGCAGGAGAGGAGGAGWGAAGG 789

**P2D8_UTR_Fw:**

CCAMTGMMTTCTGTGGTTTATGTTTTAATTTTTTTTTTTCTTTTTTCTTTCCCACCACCMCCMCGGCCSKGTGARAATAGGCTTTGTAAAAARAATTTAATTTTACGGACACWTYTCSCTAAACAGCAGCAACAGCAGCAMCAGGAGGAGCATGSGGTTKGTTTTTTTTCTTCYCGTCCTTTTTCTCGCCYGSMRSGKGGTCGGSCSKGGGAGGGGGKGAATCGGGGATACMACCCAMGGAGATGGSCMARCCTTTTTYTCWARAAAAATCCCCCCCCWTTGAAAAAGAAACGGSTACAATCAAMASMATCCCCMTCTCTGAAAAMTACASSGYSKCCMGSGCASMTCTCTCTAKMGACGGSSGCRTCTTYWCTGGGGTSAATGTRTATMATTTTACTGGGGGACCTTGTGCRRAAMTCKYGGGGYTGGGSWCTGTTGTTGTTGYGSSRGCTGGCAACCTGASTTGTRTCKYCKCGATCKSAAATGAGAACAGGGGCATCTTGAGMCCCTGYGSACRGKGCCSACAGGKGCTTCTCKATCTGYATCCTGKGATCWMAGCCMTATTGAARGACASTGATGKRCASCCSACRSCRSTTGGGATWCKYGAATTGCKSYCCTCTGGKKATGTGTGGGAGRGCTAAWGARRMACMTGCKYCTTTYWSAAAAMRAGWCGATCYCCAMCGAATGCRGGARMTYCTGATTYTAYAGGAKGCCGTGGGTKGGATTCCMCAGAMGCCAGTAACGCCTCCSGAATGGGGGATTCTCACGSTKCAGGAGRAGGARARAGSGGCCTGACAACTGTATGGAAATGAACCMATCYCCYCCTCYCCTCARTAARTGGKGGWSWATA

**Blast query x P2D8_UTR_Fw:**


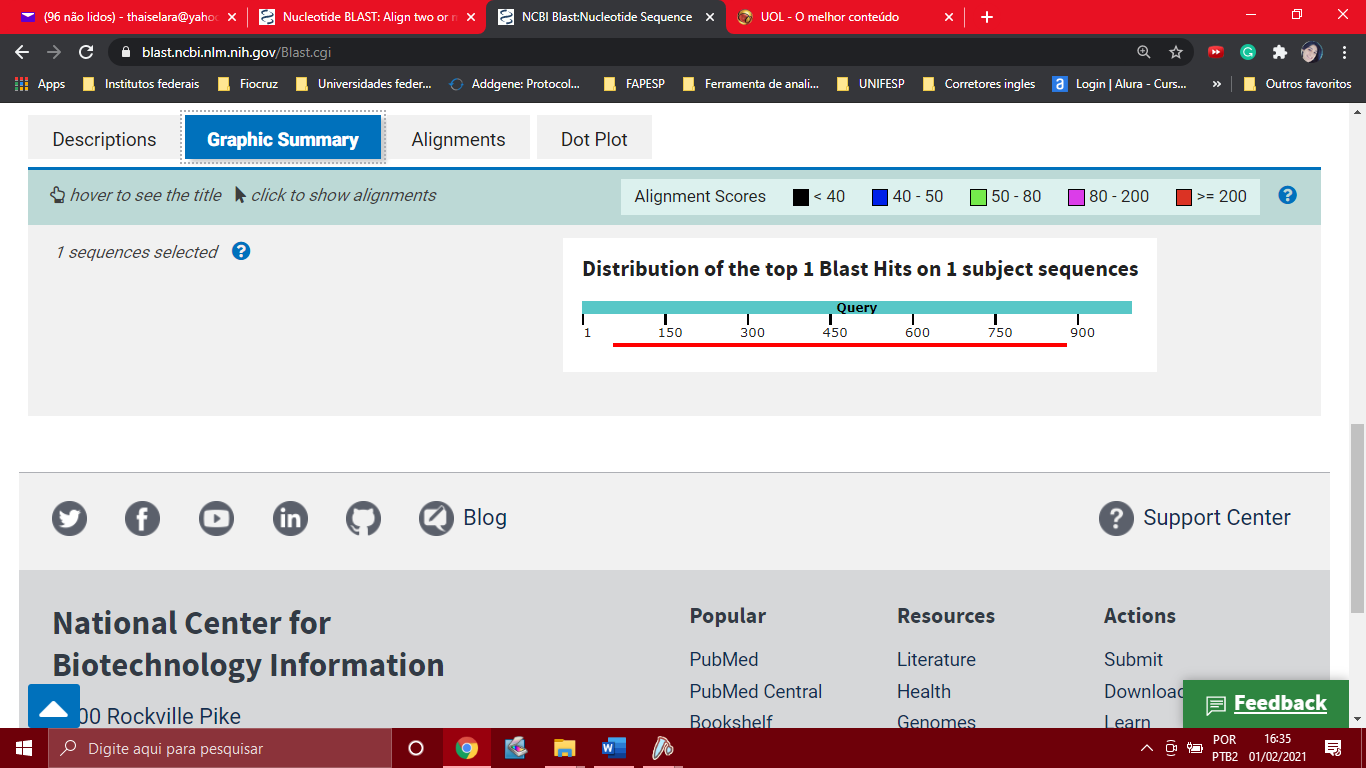


Range 1: 9 to 815

|  | | | | |  |
| --- | --- | --- | --- | --- | --- |
| Score | Expect | Identities | Gaps | Strand | |
| 643 bits(348) | 0.0 | 630/828(76%) | 30/828(3%) | Plus/Plus | |

Query 56 TTCTGTGGGTTTAT-TTTTAATCtttttttttAATCTACTTTTCCAACTCACGCTGCACC 114

|||||| ||||||| ||||||| ||||||||| ||| ||||| | ||| | | ||

Sbjct 9 TTCTGT-GGTTTATGTTTTAAT-TTTTTTTTT--TCT-TTTTTCTTTCCCAC-CACCMCC 62

Query 115 ACGGCCGTGTGAGAATAGGCTTTGTAAAAGGAATTTAATTTTACGGACACATCTCGCTAA 174

||||| |||| |||||||||||||||| ||||||||||||||||||| | || ||||

Sbjct 63 MCGGCCSKGTGARAATAGGCTTTGTAAAAARAATTTAATTTTACGGACACWTYTCSCTAA 122

Query 175 ACAGCAGCAACA--A-CAGCAGGAGGAGCATGCGGTTTGTTTTTGTTCTTCTCGTCCTTT 231

|||||||||||| | || ||||||||||||| |||| |||||| |||||| ||||||||

Sbjct 123 ACAGCAGCAACAGCAGCAMCAGGAGGAGCATGSGGTTKGTTTTTTTTCTTCYCGTCCTTT 182

Query 232 TTCTCGCCTG--CAGCGTGTCGG-CCGTGGAGGTGATGAAGCGGGGATACAACCACAAGG 288

|||||||| | | | ||||| | ||||| | ||| ||||||||| ||| || ||

Sbjct 183 TTCTCGCCYGSMRSGKG-GTCGGSCSKGGGAGGGGGKGAATCGGGGATACMACC-CAMGG 240

Query 289 AGATGGCCAAGCCTTTGTCTCAAGAAGAATCCACCCTCATTGAAAGAGCAACGGCTACAA 348

|||||| | | ||||| | || | || ||||| ||| | |||||| || ||||| |||||

Sbjct 241 AGATGGSCMARCCTTTTTYTCWARAAAAATCCCCCCCCWTTGAAAAAGAAACGGSTACAA 300

Query 349 TCAACAGCATCCCCATCTCTGAAGACTACAGCGTCGCCAGCGCAGCTCTCTCTAGCGACG 408

|||| | |||||| |||||||| | |||| | || | ||| |||||||| ||||

Sbjct 301 TCAAMASMATCCCCMTCTCTGAAAAMTACASSGYSKCCMGSGCASMTCTCTCTAKMGACG 360

Query 409 GCCGCATCTTCACTGGTGTCAATGTATATCATTTTACTGGGGGACCTTGTGCAGAACTCG 468

| || |||| |||| || ||||| ||| |||||||||||||||||||||| || ||

Sbjct 361 GSSGCRTCTTYWCTGGGGTSAATGTRTATMATTTTACTGGGGGACCTTGTGCRRAAMTCK 420

Query 469 TGGTGCTGGGCACTGCTGCTGCTGCGGCAGCTGGCAACCTGACTTGTATCGTCGCGATCG 528

|| | |||| ||| || || || | ||||||||||||| |||| || | |||||

Sbjct 421 YGGGGYTGGGSWCTGTTGTTGTTGYGSSRGCTGGCAACCTGASTTGTRTCKYCKCGATCK 480

Query 529 GAAATGAGAACAGGGGCATCTTGAGCCCCTGCGGACGGTGCCGACAGGTGCTTCTCGATC 588

|||||||||||||||||||||||| ||||| | || | ||| ||||| ||||||| |||

Sbjct 481 SAAATGAGAACAGGGGCATCTTGAGMCCCTGYGSACRGKGCCSACAGGKGCTTCTCKATC 540

Query 589 TGCATCCTGGGATCAAAGCCATAGTGAAGGACAGTGATGGACAGCCGACGGCAGTTGGGA 648

|| |||||| |||| |||| || |||| |||| ||||| || || || | ||||||

Sbjct 541 TGYATCCTGKGATCWMAGCCMTATTGAARGACASTGATGKRCASCCSACRSCRSTTGGGA 600

Query 649 TTCGTGAATTGCTGCCCTCTGGTTATGTGTGGGAGGGCTAAGTGAAGCACCTGCTTCATT 708

| | ||||||| ||||||| ||||||||||| ||||| || || ||| | ||

Sbjct 601 TWCKYGAATTGCKSYCCTCTGGKKATGTGTGGGAGRGCTAAW-GARRMACMTGCKYCTTT 659

Query 709 CAGAAAAAGAGTCGATCCCCAGCGAATGCAGGAACTCCAGATTCTACAAAGATACCGT-G 767

|||| || ||||| ||| ||||||| ||| | | |||| || | || |||| |

Sbjct 660 YWSAAAAMRAGWCGATCYCCAMCGAATGCRGGARMTYCTGATTYTAYAG-GAKGCCGTGG 718

Query 768 GTCGGATTCCACAGACGCCAGTAACACCTCG-GAATGGGGGGATTCTTCACGGCTGCAGG 826

|| ||||||| |||| ||||||||| |||| ||||||||| ||||| |||| | ||||

Sbjct 719 GTKGGATTCCMCAGAMGCCAGTAACGCCTCCSGAATGGGGG-ATTCT-CACGS-TKCAGG 775

Query 827 AGAGGAGGAGAGAAGGGGCAGCCTGACAACTGTATGGAAAAATGAACC 874

|| |||| | | | | |||||||||||||||||||| ||||||

Sbjct 776 AGR--AGGARARA-GSG---GCCTGACAACTGTATGGAAA--TGAACC 815

**P2D10_UTR_Fw:**

GGATGMWCGTMTGTASGMWTATRTYTAATTTTTTTTTTTCTTWTTTCTTTCCCACCACCACCACGGCCGTGTRARAATAGGCTTTGTAAAAARAATTTAATTTTACGGACMCWTYTCGYTAAACAGCAGCAACAGCAGCAACAGGAGGACKGSGGGTTKGGTTTTTGTTTYTTCTCGGTCCTTTTTCTCSCCTGCAGCGKGTCGGCCGKGGAGGKGGKGAATCGGGGATACAACCMCAAGGARATGGCCAAGCCTTTGTYTCAAGAARAATCCMCCCTCATTGAAAGAGCAACGGCTACAATCAACAGCATCCCCATYTYTGAARACTACAGSGTCSCCRGSGCAGYTYTYTYTAGSGACGGCCGCATCTTCACKGGKGTCAAKGTATATCATTTTACTGGGGGACCTTGKGCARAACTCGKGGKGCTGGGCAYTGYTGYTGCTGCGGCAGCTGGCAACCTGAYTTGTATCGTCSCGATCGGAAATGARAACAGGGGCATCTTGAGCCCCTGCGGACGGKGCCGACAGGTGCTTCTCGATCTGCATCCTGGGATCAAAGCCATAGTGAAGGACAGTGATGGACAGCCGACGGCAGTTGGGATTCGTGAATTGCTGCCCTCTGGTTATGTGTGGGAGGGCTAATGAAGCACCTGCTTCATTCAGAAAAAGAGTCGATCCCCAACGAATGCAGGAACTCCAGATTCTACAGGATGCCGKGGTTGGATTCCACAGACGCCAGTAACGCCTCGGATGGGGGGATTCTTCACGGCTGCAGGAGAGGAGGARAGAGGCGCTGACACTGTATGGAAAATGACACATCCTCCTCCTCCTCTACAGRYRAWTKKKGRSSGTTARATA

**Blast query x P2D10_UTR_Fw:**


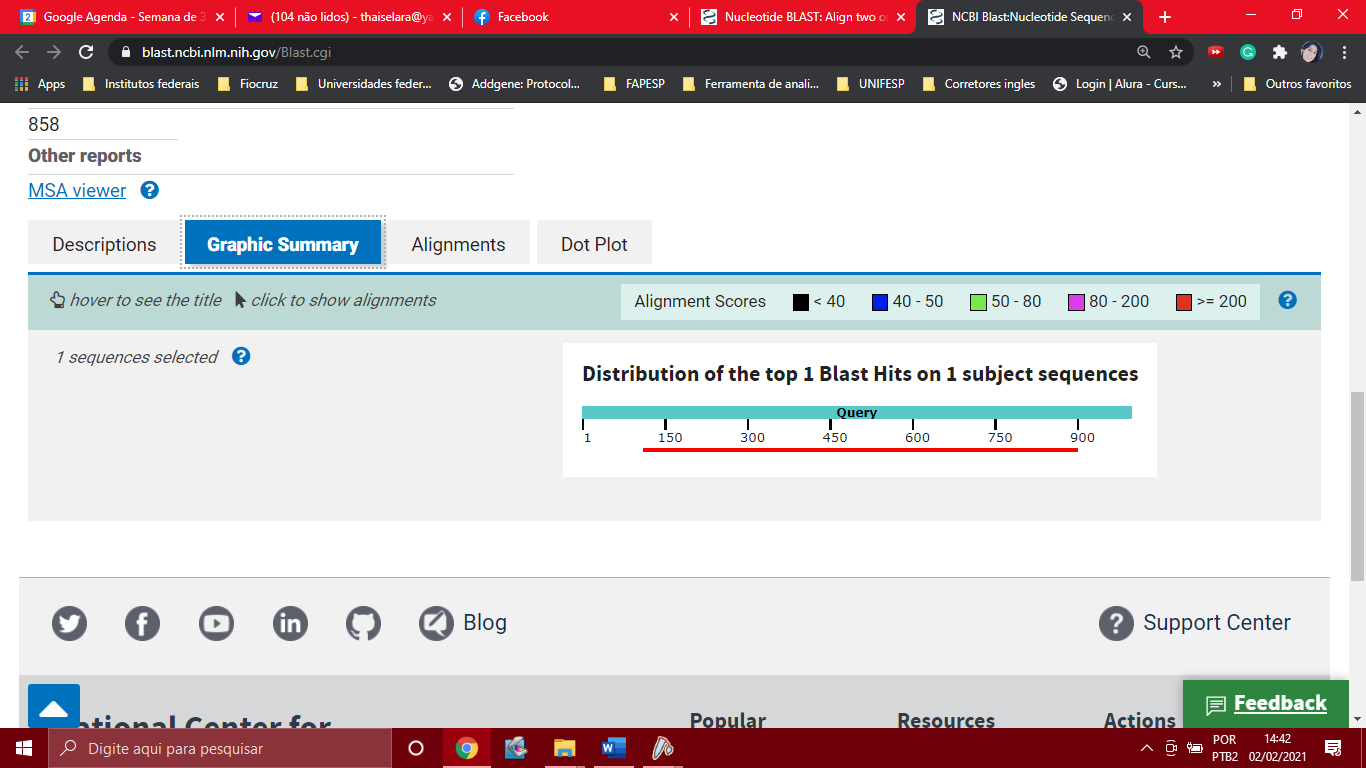


Range 1: 59 to 833

|  | | | | |
| --- | --- | --- | --- | --- |
| Score | Expect | Identities | Gaps | Strand |
| 1077 bits(583) | 0.0 | 707/788(90%) | 18/788(2%) | Plus/Plus |

Query 111 CACCACGGCCGTGTGAGAATAGGCTTTGTAAAAGGAATTTAATTTTACGGACACATCTCG 170

|||||||||||||| | |||||||||||||||| ||||||||||||||||| | | |||

Sbjct 59 CACCACGGCCGTGTRARAATAGGCTTTGTAAAAARAATTTAATTTTACGGACMCWTYTCG 118

Query 171 CTAAACAGCAGCAACAACAGCAGGAGGAGCA-TG-CGGTT-TGTTTTTG-TTCTTCTC-G 225

||||||||||||||| ||||| ||||| | | |||| ||||||| || ||||| |

Sbjct 119 YTAAACAGCAGCAACAGCAGCAACAGGAGGACKGSGGGTTKGGTTTTTGTTTYTTCTCGG 178

Query 226 TCCTTTTTCTCGCCTGCAGCGTGTCGGCCGTGGAGGTGATGAAGCGGGGATACAACCACA 285

||||||||||| ||||||||| |||||||| ||||| | ||| ||||||||||||| ||

Sbjct 179 TCCTTTTTCTCSCCTGCAGCGKGTCGGCCGKGGAGGKGGKGAATCGGGGATACAACCMCA 238

Query 286 AGGAGATGGCCAAGCCTTTGTCTCAAGAAGAATCCACCCTCATTGAAAGAGCAACGGCTA 345

|||| |||||||||||||||| ||||||| ||||| ||||||||||||||||||||||||

Sbjct 239 AGGARATGGCCAAGCCTTTGTYTCAAGAARAATCCMCCCTCATTGAAAGAGCAACGGCTA 298

Query 346 CAATCAACAGCATCCCCATCTCTGAAGACTACAGCGTCGCCAGCGCAGCTCTCTCTAGCG 405

||||||||||||||||||| | |||| ||||||| ||| || | |||| | | | ||| |

Sbjct 299 CAATCAACAGCATCCCCATYTYTGAARACTACAGSGTCSCCRGSGCAGYTYTYTYTAGSG 358

Query 406 ACGGCCGCATCTTCACTGGTGTCAATGTATATCATTTTACTGGGGGACCTTGTGCAGAAC 465

|||||||||||||||| || ||||| |||||||||||||||||||||||||| ||| |||

Sbjct 359 ACGGCCGCATCTTCACKGGKGTCAAKGTATATCATTTTACTGGGGGACCTTGKGCARAAC 418

Query 466 TCGTGGTGCTGGGCACTGCTGCTGCTGCGGCAGCTGGCAACCTGACTTGTATCGTCGCGA 525

||| || |||||||| || || ||||||||||||||||||||||| |||||||||| |||

Sbjct 419 TCGKGGKGCTGGGCAYTGYTGYTGCTGCGGCAGCTGGCAACCTGAYTTGTATCGTCSCGA 478

Query 526 TCGGAAATGAGAACAGGGGCATCTTGAGCCCCTGCGGACGGTGCCGACAGGTGCTTCTCG 585

|||||||||| |||||||||||||||||||||||||||||| ||||||||||||||||||

Sbjct 479 TCGGAAATGARAACAGGGGCATCTTGAGCCCCTGCGGACGGKGCCGACAGGTGCTTCTCG 538

Query 586 ATCTGCATCCTGGGATCAAAGCCATAGTGAAGGACAGTGATGGACAGCCGACGGCAGTTG 645

||||||||||||||||||||||||||||||||||||||||||||||||||||||||||||

Sbjct 539 ATCTGCATCCTGGGATCAAAGCCATAGTGAAGGACAGTGATGGACAGCCGACGGCAGTTG 598

Query 646 GGATTCGTGAATTGCTGCCCTCTGGTTATGTGTGGGAGGGCTAAGTGAAGCACCTGCTTC 705

|||||||||||||||||||||||||||||||||||||||||||| |||||||||||||||

Sbjct 599 GGATTCGTGAATTGCTGCCCTCTGGTTATGTGTGGGAGGGCTAA-TGAAGCACCTGCTTC 657

Query 706 ATTCAGAAAAAGAGTCGATCCCCAGCGAATGCAGGAACTCCAGATTCTACAAAGATACCG 765

|||||||||||||||||||||||| |||||||||||||||||||||||||| ||| |||

Sbjct 658 ATTCAGAAAAAGAGTCGATCCCCAACGAATGCAGGAACTCCAGATTCTACAG-GATGCCG 716

Query 766 TGGTCGGATTCCACAGACGCCAGTAACACCTCGGAATGGGGGGATTCTTCACGGCTGCAG 825

||| |||||||||||||||||||||| |||||| |||||||||||||||||||||||||

Sbjct 717 KGGTTGGATTCCACAGACGCCAGTAACGCCTCGG-ATGGGGGGATTCTTCACGGCTGCAG 775

Query 826 GAGAGGAGGAGAGAAGGGGCAGCCTGACAACTGTATGGAAAAATGAACCACTACCCTCTT 885

|||||||||| ||| || | || |||| ||||||||||||| ||| | || | |||| |

Sbjct 776 GAGAGGAGGARAGA-GG--C-GC-TGAC-ACTGTATGGAAAA-TGA-C-AC-ATCCTCCT 825

Query 886 CCTCCTCT 893

||||||||

Sbjct 826 CCTCCTCT 833

**P1C10_UTR_Rv:**

TAATTTTWGGATTTTCCTTAATCCTGCTMTSARTKWTAKGSCGCCWTCTCTCCTCCTCTCCTGCAGCCGTGAAGAATTCCCCCATTCCGAGGCGTTACTGGCGTCTGTGGAATCCAACCACGGCATCTTTGTAGAATCTGGAGTTCCTGCATTCGTTGGGGATCGACTCTTTTTCTGAATGAAGCAGGTGCTTCATTAGCCCTCCCACACATAACCAGAGGGCAGCAATTCACGAATCCCAACTGCCGTCGGCTGTCCATCACTGTCCTTCACTATGGCTTTGATCCCAGGATGCAGATCGAGAAGCACCTGTCGGCACCGTCCGCAGGGGCTCAAGATGCCCCTGTTCTCATTTCCGATCGCGACGATACAAGTCAGGTTGCCAGCTGCCGCAGCAGCAGCAGTGCCCAGCACCACGAGTTCTGCACAAKGTYYYTYWKTAAAATGATATACATTGACACCAGTGAAGATGCGGCCGTCGCTAGAGAGAGCTGCGCTGGCGACGCTGTAGTCTTCAGAGATGGGGATGCTGTTGATTGTAGCCGTTGCTCTTTCAATGAGGGTGGATTCTTCTTGAGACAAAGGCTTGGCCATCTCCTTGTGGTTGTATCCCCGMTTCACCACCTCCTCGGCCGACACGCTGCAGGCGAGAAAAAGGACGAGAARAAAMAAAACACACMGCATGCTCCTYCTGCTGCTKCTGTTGCTGCTKTTTAGCGRGATKKGTCCGTAAAATTAAATTCTTTTTACAAAGCTATTCTCMCACGTCGKKGTGGTGGTGGGAGAGAAGAAGGAAAAAAATTWTTAAAMWYCMCCSMGWTGTYGYMGCYMMWCCTCWSATGGRTGKGKGGGWKGGSAGSCA

**Reverse Complement_P1C10_UTR_Rv:**

TGSCTSCCMWCCCMCMCAYCCATSWGAGGWKKRGCKRCRACAWCKSGGKGRWKTTTAAWA

ATTTTTTTCCTTCTTCTCTCCCACCACCACMMCGACGTGKGAGAATAGCTTTGTAAAAAG

AATTTAATTTTACGGACMMATCYCGCTAAAMAGCAGCAACAGMAGCAGCAGRAGGAGCAT

GCKGTGTGTTTTKTTTYTTCTCGTCCTTTTTCTCGCCTGCAGCGTGTCGGCCGAGGAGGT

GGTGAAKCGGGGATACAACCACAAGGAGATGGCCAAGCCTTTGTCTCAAGAAGAATCCAC

CCTCATTGAAAGAGCAACGGCTACAATCAACAGCATCCCCATCTCTGAAGACTACAGCGT

CGCCAGCGCAGCTCTCTCTAGCGACGGCCGCATCTTCACTGGTGTCAATGTATATCATTT

TAMWRARRRACMTTGTGCAGAACTCGTGGTGCTGGGCACTGCTGCTGCTGCGGCAGCTGG

CAACCTGACTTGTATCGTCGCGATCGGAAATGAGAACAGGGGCATCTTGAGCCCCTGCGG

ACGGTGCCGACAGGTGCTTCTCGATCTGCATCCTGGGATCAAAGCCATAGTGAAGGACAG

TGATGGACAGCCGACGGCAGTTGGGATTCGTGAATTGCTGCCCTCTGGTTATGTGTGGGA

GGGCTAATGAAGCACCTGCTTCATTCAGAAAAAGAGTCGATCCCCAACGAATGCAGGAAC

TCCAGATTCTACAAAGATGCCGTGGTTGGATTCCACAGACGCCAGTAACGCCTCGGAATG

GGGGAATTCTTCACGGCTGCAGGAGAGGAGGAGAGAWGGCGSCMTAWMAYTSAKAGCAGG

ATTAAGGAAAATCCWAAAATTA

**Blast query x RC_P1C10 _UTR_Rv:**

| 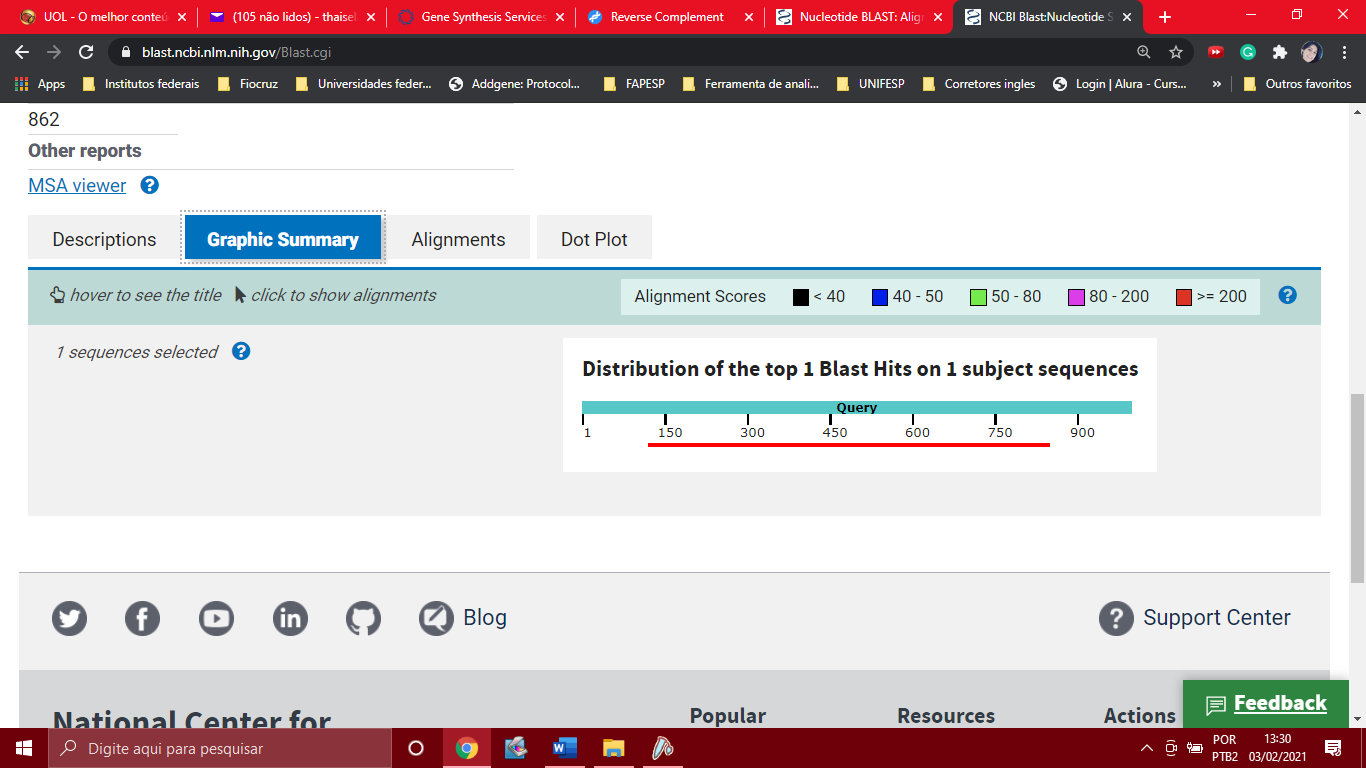  Range 1: 96 to 819 | | | | |
| --- | --- | --- | --- | --- |
| Score | Expect | Identities | Gaps | Strand |
| 1181 bits(639) | 0.0 | 692/726(95%) | 5/726(0%) | Plus/Plus |

Query 120 CGTGTGAGAATAGGCTTTGTAAAAGGAATTTAATTTTACGGACACATCTCGCTAAACAGC 179

|||| ||||||| ||||||||||| |||||||||||||||||| ||| ||||||| |||

Sbjct 96 CGTGKGAGAATA-GCTTTGTAAAAAGAATTTAATTTTACGGACMMATCYCGCTAAAMAGC 154

Query 180 AGCAACA--A-CAGCAGGAGGAGCATGCGGTTTGTTTTTGTTCTTCTCGTCCTTTTTCTC 236

||||||| | |||||| |||||||||| || |||||| || |||||||||||||||||

Sbjct 155 AGCAACAGMAGCAGCAGRAGGAGCATGCKGTGTGTTTTKTTTYTTCTCGTCCTTTTTCTC 214

Query 237 GCCTGCAGCGTGTCGGCCGTGGAGGTGATGAAGCGGGGATACAACCACAAGGAGATGGCC 296

||||||||||||||||||| ||||||| |||| |||||||||||||||||||||||||||

Sbjct 215 GCCTGCAGCGTGTCGGCCGAGGAGGTGGTGAAKCGGGGATACAACCACAAGGAGATGGCC 274

Query 297 AAGCCTTTGTCTCAAGAAGAATCCACCCTCATTGAAAGAGCAACGGCTACAATCAACAGC 356

||||||||||||||||||||||||||||||||||||||||||||||||||||||||||||

Sbjct 275 AAGCCTTTGTCTCAAGAAGAATCCACCCTCATTGAAAGAGCAACGGCTACAATCAACAGC 334

Query 357 ATCCCCATCTCTGAAGACTACAGCGTCGCCAGCGCAGCTCTCTCTAGCGACGGCCGCATC 416

||||||||||||||||||||||||||||||||||||||||||||||||||||||||||||

Sbjct 335 ATCCCCATCTCTGAAGACTACAGCGTCGCCAGCGCAGCTCTCTCTAGCGACGGCCGCATC 394

Query 417 TTCACTGGTGTCAATGTATATCATTTTACTGGGGGACCTTGTGCAGAACTCGTGGTGCTG 476

|||||||||||||||||||||||||||| || ||||||||||||||||||||||

Sbjct 395 TTCACTGGTGTCAATGTATATCATTTTAMWRARRRACMTTGTGCAGAACTCGTGGTGCTG 454

Query 477 GGCACTGCTGCTGCTGCGGCAGCTGGCAACCTGACTTGTATCGTCGCGATCGGAAATGAG 536

||||||||||||||||||||||||||||||||||||||||||||||||||||||||||||

Sbjct 455 GGCACTGCTGCTGCTGCGGCAGCTGGCAACCTGACTTGTATCGTCGCGATCGGAAATGAG 514

Query 537 AACAGGGGCATCTTGAGCCCCTGCGGACGGTGCCGACAGGTGCTTCTCGATCTGCATCCT 596

||||||||||||||||||||||||||||||||||||||||||||||||||||||||||||

Sbjct 515 AACAGGGGCATCTTGAGCCCCTGCGGACGGTGCCGACAGGTGCTTCTCGATCTGCATCCT 574

Query 597 GGGATCAAAGCCATAGTGAAGGACAGTGATGGACAGCCGACGGCAGTTGGGATTCGTGAA 656

||||||||||||||||||||||||||||||||||||||||||||||||||||||||||||

Sbjct 575 GGGATCAAAGCCATAGTGAAGGACAGTGATGGACAGCCGACGGCAGTTGGGATTCGTGAA 634

Query 657 TTGCTGCCCTCTGGTTATGTGTGGGAGGGCTAAGTGAAGCACCTGCTTCATTCAGAAAAA 716

||||||||||||||||||||||||||||||||| ||||||||||||||||||||||||||

Sbjct 635 TTGCTGCCCTCTGGTTATGTGTGGGAGGGCTAA-TGAAGCACCTGCTTCATTCAGAAAAA 693

Query 717 GAGTCGATCCCCAGCGAATGCAGGAACTCCAGATTCTACAAAGATACCGTGGTCGGATTC 776

||||||||||||| ||||||||||||||||||||||||||||||| ||||||| ||||||

Sbjct 694 GAGTCGATCCCCAACGAATGCAGGAACTCCAGATTCTACAAAGATGCCGTGGTTGGATTC 753

Query 777 CACAGACGCCAGTAACACCTCGGAATGGGGGGATTCTTCACGGCTGCAGGAGAGGAGGAG 836

|||||||||||||||| |||||||||||||| ||||||||||||||||||||||||||||

Sbjct 754 CACAGACGCCAGTAACGCCTCGGAATGGGGGAATTCTTCACGGCTGCAGGAGAGGAGGAG 813

Query 837 AGAAGG 842

||| ||

Sbjct 814 AGAWGG 819
